# Community context and pCO_2_ impact the transcriptome of the “helper” bacterium *Alteromonas* in co-culture with picocyanobacteria

**DOI:** 10.1038/s43705-022-00197-2

**Published:** 2022-11-15

**Authors:** Marcelo Malisano Barreto Filho, Zhiying Lu, Melissa Walker, J. Jeffrey Morris

**Affiliations:** University of Alabama at Birmingham Department of Biology, 1300 University Blvd CH464, Birmingham, AL 35294 USA

**Keywords:** Water microbiology, Microbial biooceanography

## Abstract

Many microbial photoautotrophs depend on heterotrophic bacteria for accomplishing essential functions. Environmental changes, however, could alter or eliminate such interactions. We investigated the effects of changing pCO_2_ on gene transcription in co-cultures of 3 strains of picocyanobacteria (*Synechococcus* strains CC9311 and WH8102 and *Prochlorococcus* strain MIT9312) paired with the ‘helper’ bacterium *Alteromonas macleodii* EZ55. Co-culture with cyanobacteria resulted in a much higher number of up- and down-regulated genes in EZ55 than pCO_2_ by itself. Pathway analysis revealed significantly different transcription of genes involved in carbohydrate metabolism, stress response, and chemotaxis, with different patterns of up- or down-regulation in co-culture with different cyanobacterial strains. Gene transcription patterns of organic and inorganic nutrient transporter and catabolism genes in EZ55 suggested resources available in the culture media were altered under elevated (800 ppm) pCO_2_ conditions. Altogether, changing transcription patterns were consistent with the possibility that the composition of cyanobacterial excretions changed under the two pCO_2_ regimes, causing extensive ecophysiological changes in both members of the co-cultures. Additionally, significant downregulation of oxidative stress genes in MIT9312/EZ55 cocultures at 800 ppm pCO_2_ were consistent with a link between the predicted reduced availability of photorespiratory byproducts (i.e., glycolate/2PG) under this condition and observed reductions in internal oxidative stress loads for EZ55, providing a possible explanation for the previously observed lack of “help” provided by EZ55 to MIT9312 under elevated pCO_2_. If similar broad alterations in microbial ecophysiology occur in the ocean as atmospheric pCO_2_ increases, they could lead to substantially altered ecosystem functioning and community composition.

## Introduction

Driven largely by anthropogenic activities, the carbon dioxide content of Earth’s atmosphere (pCO_2_) is increasing at a rate unprecedented in history [1]. One impact of this change is ocean acidification caused by the absorption of CO_2_ by seawater [2]. The rates of these changes are influenced by marine phytoplankton, which are responsible for roughly half of global primary productivity [3, 4]. Phytoplankton are in turn metabolically interconnected with bacterioplankton via the release of dissolved organic matter (DOM) which is remineralized via aerobic respiration, creating an internal carbon cycle known as the microbial loop [5]. The relative rates of carbon fixation and carbon release, as well as carbon export to sediments or higher trophic levels, influence the overall pace of pCO_2_ change [6]. As a result, substantial effort has been directed toward understanding how changing pCO_2_ will affect phytoplankton growth dynamics [7, 8].

The picocyanobacteria *Prochlorococcus* and *Synechococcus* are the two most abundant phytoplankton genera in the open ocean and are thus important components of the marine carbon cycle [9]. Climate change models considering only increased temperature and light have predicted significant increases in cell numbers for both taxa by year 2100 [9]. However, the two genera responded differently in culture-based experiments to future pCO_2_ and temperature [10], with *Prochlorococcus* showing substantially reduced growth rates under projected year 2100 pCO_2_ (800 ppm) [11]. Models incorporating phytoplankton response to both temperature and pCO_2_ suggested that *Prochlorococcus* would be outcompeted throughout its range by *Synechococcus* [12] with potentially dramatic impacts on oceanic carbon cycling.

Interestingly, *Prochlorococcus’* growth impairment at high pCO_2_ was partially caused by transcription changes in the “helper” bacterium *Alteromonas macleodii* EZ55 with which it was co-cultured in these experiments [7]. Previous experiments showed that *Prochlorococcus* cultures depended on helper bacteria like EZ55 to tolerate H_2_O_2_ found in culture media [13, 14]. However, EZ55 downregulated the H_2_O_2_-removing enzyme catalase at 800 ppm pCO_2_, effectively withholding help from *Prochlorococcus* and leading to reduced growth rates and elevated mortality [7].

The helper interaction between EZ55 and *Prochlorococcus* represents only one of many similar exchanges between marine microbes. For instance, many “Black Queen” (BQ) interactions involving leaky biological functions whose products are available to other community members [15, 16] create evolutionary incentives for microbes to become dependent on each other through metabolic streamlining. BQ evolutionary outcomes range from decreased gene expression [17] to gene loss [14, 16] and produce “beneficiary” organisms such as *Prochlorococcus* that are dependent on “helper” organisms like *Alteromonas* to perform leaky functions [14, 18, 19]. Because any of these interactions may be disrupted by elevated pCO_2_ in the same way as was observed between *Prochlorococcus* and EZ55, it is possible that carbon cycling in future oceans may experience changes that are difficult to predict based on the behavior of axenic cultures.

Our ability to assess the impact of future pCO_2_ on marine communities is thus limited by the reduced ecological complexity of laboratory experiments [2], and in particular microalgal experiments using axenic cultures can misrepresent marine communities’ natural dynamics [20]. Therefore, studies using simplified communities comprising different functional groups of phytoplankton and heterotrophic bacteria provide a more realistic approach to understanding how carbon cycle dynamics will be affected by global change. Here we explored the impact of projected year 2100 pCO_2_ (800 ppm) on transcription in co-cultures of *Alteromonas* sp. EZ55 with the picocyanobacterial *Prochlorococcus* MIT9312, *Synechococcus* sp. CC9311, and *Synechococcus* sp. WH8102. Our goal was both to understand how these organisms respond to pCO_2_ change as well as how EZ55 responds to the presence of different cyanobacterial partners. Our results demonstrated that year 2100 pCO_2_ will likely impact metabolic conversations between cyanobacteria and marine bacteria by altering the availability of secreted metabolites and inorganic nutrients, with secondary effects on stress physiology, all of which impact community ecological function.

## Methods

### Strains

Six clones each of the open ocean *Synechococcus* strain WH8102 and the coastal *Synechococcus* strain CC9311 were obtained by dilution to extinction in SN media [21]. The parent cultures of each organism were obtained from the National Center for Marine Algae (Boothbay Harbor, Maine) and were axenic upon receipt. Six clones of *Alteromonas* sp. strain EZ55 and *Prochlorococcus* MIT9312 were also previously obtained and cryopreserved at −80 °C [7]. The EZ55 clones used in our *Synechococcus* co-cultures were the same 6 clones used in our previous transcriptomic study of MIT9312 [7] in order to maximize the comparability of results between that study and the present study. Co-cultures were initiated by mixing each of the six clones of CC9311 and WH8102 with one of the EZ55 clones.

### Culture conditions

*Synechococcus* cultures were grown under similar conditions to those described in our previous experiment with *Prochlorococcus* [7]. Briefly, all cultures were prepared in acid-washed conical-bottom glass centrifuge tubes containing 13 mL of artificial seawater (ASW) amended with nutrient stocks [21] and with acid and/or base to control pCO_2_. ASW (per L: 28.41 g NaCl, 0.79 g KCl, 1.58 g CaCl2*2H2O, 7.21 g MgSO4*7H2O, 5.18 g MgCl2*6H2O) was sterilized in acid-washed glass bottles, amended with 2.325 mM (final concentration) of filter-sterilized sodium bicarbonate, then bubbled with sterile air overnight. *Synechococcus* cultures were grown in SEv (per L: 32 μM NaNO_3_, 2 μM NaH_2_PO_4_, 20 μL SN trace metal stock, and 20 μL F/2 vitamin stock). The primary differences between this medium and the PEv medium used in our earlier *Prochlorococcus* study are the nitrogen source (NO_3_^−^ vs. NH_4_^+^, with molar concentration of N and N:P ratios identical to PEv) and the addition of F/2 vitamins [21]. Carbonate chemistry of each media batch was determined prior to pCO_2_ manipulations by measuring alkalinity and pH by titration and colorimetry, respectively [7, 11] and then using the *oa* function in *seacarb* package in R to determine how much hydrochloric acid and bicarbonate (for 800 ppm pCO_2_) or sodium hydroxide (for 400 ppm pCO_2_) was needed to achieve desired experimental conditions [22]. Acid and base amendments were introduced immediately prior to inoculation. Cultures were grown in a Percival growth chamber at 21 °C under 150 μmol photons m^−2^ s^−1^ on a 14:10 light:dark cycle. *Synechococcus* cultures were grown on a rotating tissue culture wheel at approximately 60 rpm.

### Growth experiments

The transcriptomes of all six clonal replicates of each *Synechococcus* strain along with their EZ55 partners were assessed under approximately 400 (based on atmospheric pCO_2_ measured at Mauna Loa in 2015, when the experiment was planned) or 800 ppm (i.e., approximate predicted year 2100 pCO_2_ under IPCC scenario A2) pCO_2_. Prior to RNA extraction, each culture was acclimated to experimental conditions for three transfer cycles (approximately 14 generations). Growth was tracked by flow cytometry using a Guava HT1 Flow Cytometer (Luminex Corporation, Austin, TX). EZ55 cell concentrations were determined by dilution onto YTSS agar (per L, 4 g tryptone, 2.5 g yeast extract, 15 g sea salts, 15 g agar). Whenever *Synechococcus* cell densities reached 2.6 × 10^5^ cells mL^−1^, cultures were diluted 26-fold into fresh media. Preliminary experiments revealed that this cell concentration was low enough that growth was not limited by nutrients and pH and pCO_2_ were not significantly impacted by cyanobacterial carbon concentrating mechanisms. In the final transfer cycle, each culture was split into 5 identical subcultures to increase the biomass available for RNA extraction; all 5 subcultures of each clone were then pooled and collected on a single 0.2 μm polycarbonate filter by gentle syringe filtration, then flash-frozen in liquid nitrogen and stored at −80 °C prior to RNA extraction. For WH8102 cultures, an average of 4.04 × 10^7^ WH8102 cells and 3.91 × 10^8^ EZ55 cells were collected per filter, and for CC9311 cultures, an average of 5.47 × 10^7^ CC9311 and 7.33 × 10^8^ EZ55 cells were collected per filter. Carbonate chemistry parameters for the cultures are shown in Table 1.

**Table 1:** Carbonate chemistry for *Synechococcus* cultures.

		400 ppm			800 ppm		
Strain	Salinity	Alkalinity	pH	pCO2	Alkalinity	pH	pCO2
CC9311	34	2398	8.09 ± 0.010	370	2317	7.79 ± 0.008	806
WH8102	32.5	2372	8.04 ± 0.04	437	2312	7.76 ± 0.017	867

Malthusian and exponential growth rates, lag durations, and post-transfer die-offs for cyanobacteria were calculated as described previously [7]. The effect of pCO_2_ on these properties was analyzed using linear mixed-effects models in R using the *lme4* package, followed by post-hoc contrasts using *emmeans*.

### RNA library preparation and sequencing

RNA extraction was performed separately for each replicate culture with the RNeasy Mini Kit (Qiagen, Valencia, CA, USA) with a small modification of the lysis step [7]. rRNA was removed with the Ribo-Zero rRNA Removal Kit for Bacteria (Illumina, San Diego, CA, USA) [7]. Following rRNA removal, samples were purified and concentrated with a RNeasy MiniElute cleanup kit (Qiagen). Quantity and quality of post-digestion RNA were assessed with an Agilent 2100 Bioanalyzer (Agilent, Santa Clara, CA, USA). mRNA library preparation for Illumina Hi-seq 2500 paired-end sequencing (PE100) used TruSeq RNA sample prep kit v2 (Illumina, San Diego, CA, USA). DNA fragment length was 100 bp, paired ends were non-overlapping, and the insert size was approximately 300 bp. Individual barcode sequences were added to sequence reads for multiplex sequencing which were run in a single lane at the Sulzberger Columbia University Genome Center (CUGC) (New York, NY, USA). Our sequences files are accessible from NCBI (BioProject PRJNA377729, SRA accession numbers SRX2619948-SRX2619957, SRX3033334-SRX3033345, and SRX14411251-SRX14411274).

### Gene transcription analysis

The reads obtained from this study as well as all reads from our previous experiment with MIT9312 and EZ55 [7] were analyzed together here. Sequence reads (i.e., per-cycle BCL basecall files) were translated into per-read FASTQ files for downstream analysis at the sequencing facility with the software bl2fastq using default settings along with adaptor trimming to remove barcodes. We followed a slightly modified version of the workflow described in [23] for alignment counting and differential gene transcription using the packages *Rsubread* and *edgeR* in R v4.02 [24]. The distribution of FASTQ quality scores were assessed with the *qualityScores* function. Cyanobacterial reference genomes were obtained from Ensemble Bacteria (ASM1264v1, ASM1458v1, ASM19597v1, respectively for MIT9312, CC9311 and WH8102). EZ55 data was obtained from IMG/M (taxon ID 2785510739); this complete genome assembly [25] was a superior version compared to the one used in our previous analysis of MIT9312 [7]. Genome indices were built prior to alignment using the function *buildindex*. Subsequently, reads were aligned to indices using the default settings of the *align* function. The resulting BAM files were counted against cyanobacterial and EZ55 annotated genomes using the *featurecounts* function.

We estimated differential gene transcription (DGE) probabilities and fold change of transcript counts using *edgeR*. Prior to DGE analysis, counts of genes with low transcription levels were filtered using the *filterByExpr* function, and remaining counts were normalized using the function *calcNormFactors* to eliminate composition biases between libraries. General linear models estimated common, trend, and tag-wise dispersions and the impacts of different experimental design parameters on normalized counts (*glmFIT* function). For cyanobacteria, a design matrix was prepared to test for DGE between the two pCO_2_ experimental conditions. For *Alteromonas* EZ55, our design matrix tested for the effects of coculture and pCO_2_, as well as the interaction between those two factors. Statistical analysis was performed based on our fitted model and the following custom contrasts were applied using the function *makeContrasts:* (i) the effect of treatment (i.e., 800 ppm Vs 400 pCO_2_); (ii) the general effect of coculture averaged across all partners; (iii) the effect of specific cyanobacteria *in addition* to the general co-culture response; (iv) the general interaction between co-culture and pCO_2_; and (v) the interaction between specific cyanobacteria and pCO_2_ *in addition* to the general interaction term. Significant DGE was defined as genes having unadjusted *p* values < 0.05 and log fold change (logFC) > 1 in pairwise comparisons within our model. DGE genes for cyanobacteria were tested for Gene Set Enrichment Analysis (GSEA) using the function *gseKEGG* in the package *clusterProfiler*. Transcription levels for EZ55 were analyzed using Over Representation Analysis (ORA) with the function *enrichKEGG* in *clusterProfiler* [26].

### EZ55 growth experiments with photorespiration metabolites

We investigated the ability of EZ55 to grow on metabolic intermediates in the photorespiration pathway as their sole carbon source. Glycine, glycolate, glucose, and glyoxylate stock solutions (concentrations of 20%, 4%, 4% and 10% W/V, respectively) were filter sterilized using a 0.2 μM filter. The pH of glycolate and glyoxylate stocks was adjusted to approximately 7 using 10 M NaOH. EZ55 clones were inoculated into ASW supplemented with Pro99 nutrients [21] and 0.1% (W/V) glucose [25] and acclimated for 24 hours at 28 °C with orbital shaking at 120 rpm. After acclimation, cultures were diluted 1000-fold into 10 mL Pro99 (without glucose) for a further 24 h cultivation under the same cultivation conditions, then 5 μL of each culture were added into duplicate wells of a UV-sterilized 96-well transparent plate containing 215 μL Pro99 media with either glycine, glycolate, glyoxylate, or glucose at 0.1% concentration as carbon sources, or else no added carbon (as a negative control). The plate was sealed with UV sterilized transparent sealing film and cultivated in a Synergy H1 plate reader (Biotek, Vermont, USA) at 28 °C for 48 h in continuous reading mode, collecting 600 nm wavelength optical density at 5 min intervals. A culture’s exponential growth rate was determined as the maximum slope of its growth curve, determined by linear regressions of OD vs. time in a sliding time point window [27].

The detection of intracellular H_2_O_2_ was performed according to Lu et al. [28], with slight modification. Briefly, 1.5 ml of culture was centrifuged at 8000 rpm for 5 min, the supernatant was removed, and the pellet was resuspended in 1 ml phosphate buffered saline (pH = 7.4, Fisher). 5 μl of 1 mM 2′,7′-dichlorodihydrofluorescein diacetate was added to the resuspension and vortexed for 5 seconds and then incubated for 1 h on a shaker (120 rpm) in the dark. The suspension was centrifuged at 8000 rpm for 5 min, the pellet was washed twice with PBS, and finally resuspended in 200 μl PBS. Fluorescence was measured by flow cytometry at excitation/emission wavelengths of 485/535 nm. The H_2_O_2_ level for a sample was estimated as the mean of log fluorescence at 535 nm for 10,000 events.

### Detection of glycolate utilization genes

Several genes involved in the bacterial glycolate utilization pathway (glycolate/lactate oxidase, the 3 subunits of glycolate dehydrogenase, and tartronate semialdehyde reductase) were not annotated in the reference genomes for our organisms so we specifically sought to detect them using a reciprocal BLAST analysis. We retrieved any sequences from each of the four reference genomes with high similarity (*E*-value < 0.001) to the relevant genes from *Escherichia coli* and/or *Synechococcus elongatus* using blastp [29] and then back-matched each retrieved sequence to the *E. coli* or *S. elongatus* reference genome. If the reciprocal match was the same gene used in the original BLAST search, we considered the match significant. Retrieved sequences were aligned using MUSCLE 3.8.425 [30] and visualized in MVIEW via the EMBL-EBI web interface [31]. Bootstrapped maximum likelihood trees of GlcDF and GOX/LOX sequences were created in MEGA version 11; model parameters for tree construction were chosen based on the combination that minimized the Bayesian Information Criterion for model fit for each dataset using MEGA’s model selection tool [32].

## Results

### Growth dynamics of *Synechococcus* strains

The growth of *Synechococcus* CC9311 was not significantly impacted by pCO_2_ conditions in co-culture with *Alteromonas* EZ55, whereas *Synechococcus* WH8102 experienced similar, though less severe, growth reductions to those we observed previously in *Prochlorococcus* MIT9312 [7] (Fig. S1). WH8102 actually had a significantly elevated exponential growth rate under 800 ppm pCO_2_ (~11% increase based on a linear mixed-effects model, *p* = 0.009), but significantly increased lag phase duration (2.1 ± 0.5 d, linear mixed-effects model, *p* < 0.001) and post-transfer cell death (7.8 ± 6.5 % loss, linear mixed-effects model, *p* = 0.001) reversed this growth rate increase and led to a significantly reduced realized, or Malthusian, growth rate (~17% reduction, linear mixed-effects model, *p* = 0.006). We did not observe the growth of axenic *Synechococcus* strains for this study, so we cannot address whether or not these differences reflect a change in “helping” behavior of EZ55, as was observed for MIT9312 [7].

### Cyanobacterial responses to elevated pCO_2_

**Fig. 1:**
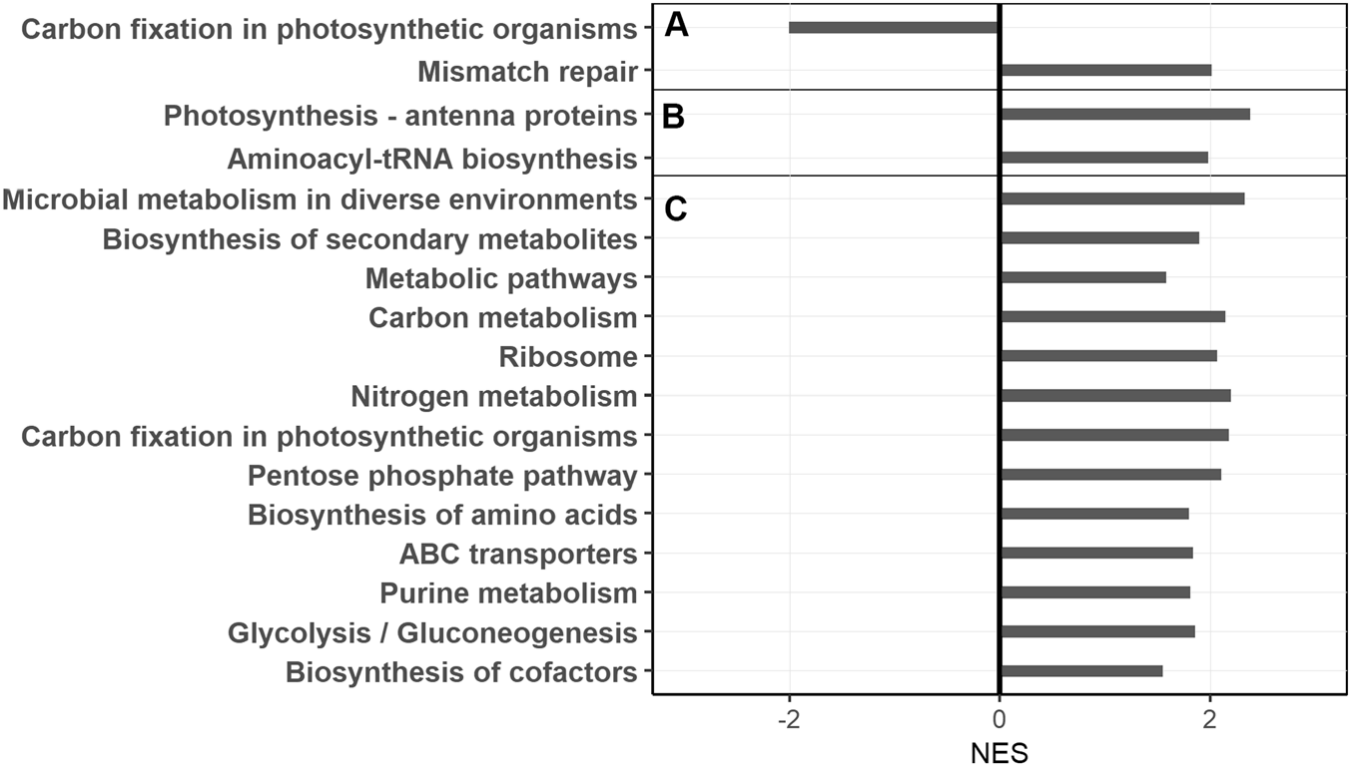
Gene set enrichment analysis. Genes were binned into sets using KEGG annotations for *Prochlorococcus* MIT9312 (**A**), *Synechococcus* WH8102 (**B**) and *Synechococcus* CC9311 (**C**) representing categories differentially regulated between 400 and 800 ppm pCO_2_. Normalized enrichment scores (NES) indicate the distribution of KEGG categories across a list of genes ranked by hypergeometrical score (HGS). Higher enrichment scores indicate a shift of genes belonging to the indicated KEGG category towards either end of the ranked list representing up or down regulation (positive or negative values, respectively).

MIT9312, CC9311, and WH8102 had 37, 30 and 13 genes significantly (logFC > 1, *p* < 0.05) differentially transcribed between pCO_2_ treatments (Fig. S2, Table S1). Consistent with our previous report using a different pipeline on the same sequences [7], MIT9312 decreased transcription of carboxysome shell genes, RUBISCO genes, and several high-light inducible (HLI) genes, and increased transcription of the spectrin repeat “co-culture response gene” [33] (Fig. S3A). In contrast, WH8102 increased transcription of RUBISCO and carboxysome genes (Fig. S3B) as well as several N-acquisition genes. Like MIT9312, CC9311 downregulated HLI genes as well as other stress-related genes (Fig. S3C). GSEA confirmed the downregulation of carbon fixation pathways in MIT9312 (Fig. 1A) and the upregulation of carbon fixation, nutrient acquisition, and other catabolic and anabolic pathways in WH8102 (Fig. 1B). MIT9312 also increased transcription of its DNA mismatch repair system under elevated pCO_2_. CC9311 upregulated photosynthesis antenna protein synthesis and aminoacyl-tRNA biosynthesis under elevated pCO_2_ (Fig. 1C).

### *Alteromonas* sp. EZ55 response to elevated pCO_2_

Many more genes were significantly differentially regulated under 800 ppm pCO_2_ in EZ55 relative to any of the cyanobacteria tested (Table S2, Fig. S4A). Genes for amino acid and peptidoglycan biosynthesis as well as catabolism of diverse sugars were over-represented (Fig. S5A). All statistically differentially transcribed genes in the three most over-represented KEGG categories were downregulated. In contrast, the starch and sucrose catabolism category revealed that two α-amylase glycoside hydrolase enzymes, sucrose phosphorylase and amylosucrase, were transcribed more at 800 ppm.

### EZ55 general response to co-culture

We were also able to compare axenic EZ55 transcriptomes to those in co-culture with cyanobacteria. The impact of co-culture on EZ55 was much more pronounced than that of pCO_2_. Remarkably, 1144 genes were significantly differentially transcribed in co-culture with cyanobacteria relative to axenic EZ55 (Fig. S4B, Table S3). Additionally, the pCO_2_ response was significantly modulated by co-culture for 457 genes (Fig. S4C, Table S4). More overrepresented KEGG pathways were identified for the co-culture response (Fig. S5B) and the interaction of co-culture with pCO_2_ (Fig. S5C) than for pCO_2_ alone (Fig. S5A).

**Fig. 2:**
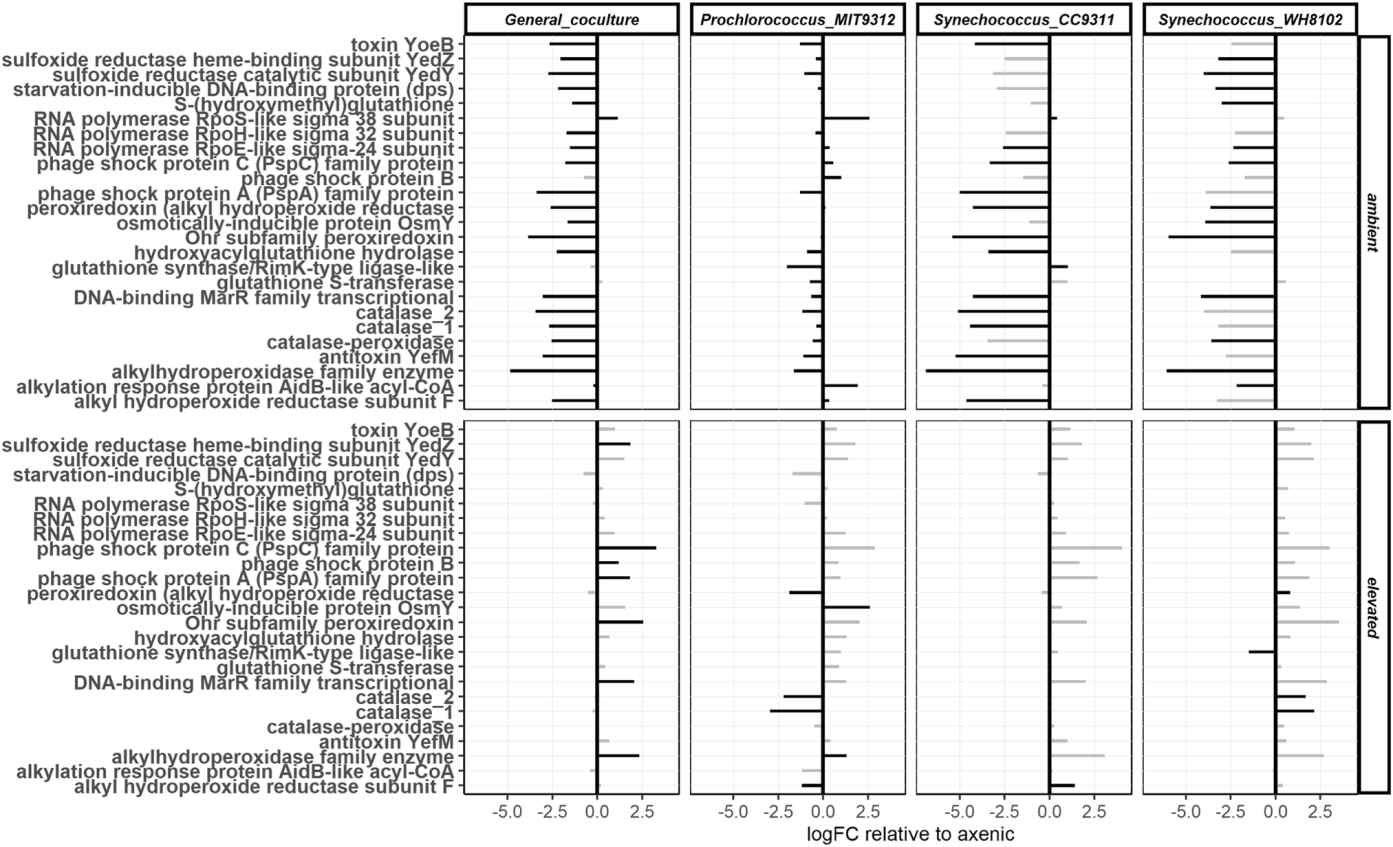
Summary plot of stress-related gene products in *Alteromonas* EZ55. Plots represent the general response to co-culture (column 1), and co-culture with specific cyanobacteria (columns 2-4) at either 400 or 800 ppm pCO_2_, relative to transcription under axenic conditions at the same pCO_2_. Log_2_ fold change (logFC) is plotted on the *x*-axis relative to axenic *Alteromonas* under the same pCO_2_ condition. Differentially transcribed genes (*p* < 0.05 and logFC > 1) are highlighted in black. Black bars in column 1 indicate the average co-culture response is significantly different from the axenic response at the same pCO_2_; black bars in columns 2-4 indicate significant difference between the specific cyanobacterial response and the general coculture response shown in column 1.

Overall, transcripts for differentially regulated stress-related genes were depleted relative to axenic EZ55 at 400 ppm pCO_2_, with the sole exception of the RNA polymerase stationary-phase sigma factor (*rpoS*) (Fig. 2; Table S5). These genes included the antioxidant enzymes catalase, catalase-peroxidase, and alkyl hydroperoxide reductase. Co-cultures at 400 ppm upregulated transcripts for transporters of N, P, Fe, organic acids (e.g., glycolate, acetate, lactate), and a wide variety of carbon substrates (Fig. S6; Table S6). On the other hand, at 800 ppm pCO_2_, most stress genes were upregulated in co-culture, and most transporters had unchanged transcription relative to axenic EZ55.

Transcripts for most chemotaxis genes were more abundant in co-culture relative to axenic at 400 ppm but generally unaffected or downregulated in co-culture at 800 ppm (Fig. S7; Table S7). On the other hand, flagella-related genes were mostly downregulated in co-cultures at 400 ppm but upregulated at 800 ppm. Many central carbon metabolism genes were also differentially regulated, with enzymes involved in glyoxylate/dicarboxylate, propanoate, pyruvate, and amino acid catabolism highly overrepresented (Fig. S8; Table S8). Most differentially transcribed central metabolism transcripts were enriched in co-culture at 400 ppm, except for genes encoding enzymes in the methylcitrate cycle and glycine betaine synthesis from choline, which were downregulated. At 800 ppm, however, only two central metabolism enzymes were differentially transcribed in co-culture, and both were depleted relative to axenic growth.

### The effect of specific cyanobacterial partners on EZ55 gene transcription

The previous section analyzed the general co-culture response of EZ55, but our analysis also revealed many genes that were differentially transcribed depending on which cyanobacterial partner EZ55 was cultured with (Fig. S4, panels D-F; Tables S9, S10, S11). For many of these, the strain-specific co-culture response was also different between pCO_2_ treatments (Fig. S4, panels G-I, Tables S12, S13, S14). Similar KEGG categories were overrepresented in EZ55’s response to co-culture with specific cyanobacteria compared to co-culture in general (Fig. S5, panels D-I), suggesting that these species-specific differences reflected tailoring of the same general pathways rather than fundamental differences in metabolic response. In contrast, there were fewer significant species-specific differences in gene transcription relative to the general co-culture response at 800 ppm. One notable difference was that at 400 ppm most EZ55 stress-related genes were more highly transcribed with MIT9312 than with either *Synechococcus* strain (Fig. 2). Moreover, the genes encoding two EZ55 catalases as well as alkyl hydroperoxide reductase were decreased at 800 ppm in co-culture with MIT9312 but increased with WH8102, and unchanged relative to the general co-culture response for CC9311.

Transcription of some nutrient acquisition genes was also strain-specific (Fig. S6), and like with stress genes, showed markedly different patterns for co-cultures with MIT9312 compared to the *Synechococcus* strains. For example, at 400 ppm the EZ55 NH_4_^+^ transporter was upregulated in co-culture with MIT9312 and WH8102, but at 800 ppm NH_4_^+^ transporter transcripts substantially declined in co-culture with MIT9312 but not with either *Synechococcus* strain. A few EZ55 organic carbon transporters also shifted transcription in response to pCO_2_ in a species-specific manner. For example, at 400 ppm_,_ transcripts encoding two fucose permeases were upregulated in co-culture with MIT9312 (although a third was downregulated), and an acetate transporter was more highly transcribed with WH8102. B-vitamin transport genes were upregulated in co-culture with MIT9312 under 400 ppm and with WH8102 under 800 ppm. The species-specific transcription patterns of chemotaxis and flagellar genes were similar to the trends observed for the general co-culture response, although the magnitudes of the shifts varied significantly between strains in many cases (Fig. S7).

At 400 ppm pCO_2_, many central metabolism genes were differently transcribed in EZ55 relative to axenic culture depending on the specific cyanobacterial partner (Figs. S8, 3, S9). For example, EZ55 co-cultured with both *Synechococcus* strains increased transcription of genes involved in the glycine cleavage system (GCS) although the magnitude of change was significant only when paired with CC9311; in contrast, these genes were downregulated in co-culture with MIT9312. The bacterial glyoxylate shunt enzymes, malate synthase and isocitrate lyase, whose metabolites overlap with GCS, were upregulated in co-culture with WH8102. EZ55 co-cultured with *Synechococcus* strains but not with MIT9312 increased transcription of valine and acetate catabolism genes and decreased transcription of glycine betaine synthesis genes. EZ55 paired with MIT9312 increased transcription of genes encoding pyruvate dehydrogenase. Beta-oxidation of fatty acids was generally upregulated at 400 ppm but downregulated at 800 ppm. Glycine betaine metabolism was downregulated for EZ55 co-cultures with CC9311 at 400 ppm but upregulated at 800 ppm. Overall, there were fewer species-specific regulatory changes relative to axenic growth at 800 ppm pCO_2_ than at 400 ppm.

**Fig. 3:**
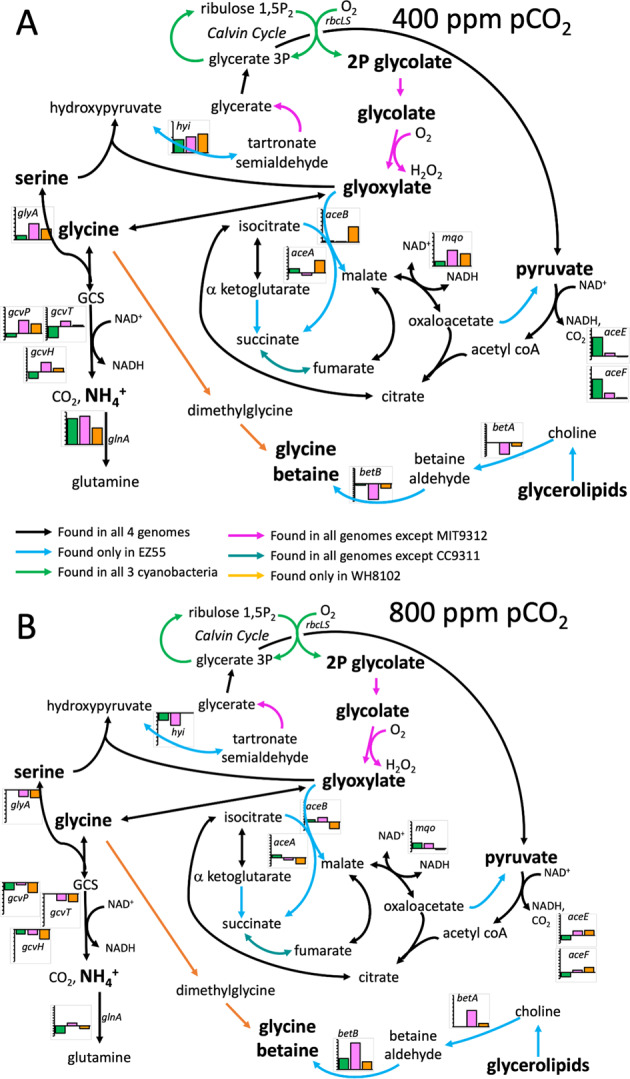
Reconstruction of metabolic pathways related to the recycling of photorespiratory byproducts whose genes were significantly differently regulated in *Alteromonas* EZ55 between axenic and co-culture conditions at the indicated CO_2_ partial pressure. Panel **A**, 400 ppm pCO2; Panel **B**, 800 ppm pCO2. Small inset graphs indicate the log fold change of transcription, relative to axenic culture under the same pCO_2_ condition, in co-culture with MIT9312 (green bars), CC9311 (pink bars), or WH8102 (orange bars). Gene names correspond to the related genes in *E. coli*. Only genes that were differently transcribed between axenic and co-culture in at least one condition are shown; not all bars represent significant differences, and specific statistical tests for all gene products are shown in Fig. S8. Metabolites in bold print are hypothesized to be exudates exchanged between co-culture constituents under certain conditions. Arrow colors indicate which of the four genomes are capable of performing the indicated reaction based on annotated gene functions and our own phylogenetic analyses (Figs. S10–S13); see inset legend for explanation.

### Metabolism of photorespiration by-products by EZ55

We investigated the possibility that EZ55 may be capable of metabolizing the byproducts of photorespiration, which may be less abundant at 800 ppm pCO_2_ and may be obligately secreted by cyanobacteria. First, we reconstructed the glycolate catabolism pathways in all four organisms from genomic information (Fig. 3). To detect possibly misannotated genes for gaps in pathways, we performed reciprocal BLAST queries using amino acid sequences for missing genes from *Escherichia coli* and/or *Synechococcus elongatus* (Fig. S10). First, we confirmed that there was no analog for 2PG phosphatase in the MIT9312 genome. We discovered candidates for tartronate semialdehyde reductase in EZ55 and both *Synechococcus* strains, but no hydroxypyruvate isomerase for *Synechococcus* or glyoxylate carboligase candidates for any organism. We discovered candidates for *glcD*, one of the subunits of glycolate dehydrogenase, in all four genomes, but only the two *Synechococcus* strains had matches for the other two subunits *glcE* and *glcF*. Because all three genes are required to function in *E. coli* [34], we investigated the phylogenies of the *glcD* candidates in EZ55 and MIT9312 more closely. The MIT9312 *glcD* fell into a clade containing the alternative *glcD* discovered in *Synechocystis* PCC6803 [35], and versions of this *glcD2* were also found in both *Synechococcus* genomes studied here (Fig. S11). However, it is not clear if this enzyme can catalyze the conversion of glycolate to glyoxylate without the assistance of the *glcEF* subunits [35]. The EZ55 protein, on the other hand, was almost twice as long as the *E. coli* GlcD, and we discovered that the long C-terminal extension was quite similar to the *E. coli* GlcF Fe-S protein, suggesting that this novel large protein may be capable of catalyzing the reaction performed by the three *glc* subunits by itself. We also discovered that this large GlcDF fusion protein was conserved throughout the marine Gammaproteobacteria, but not in *E. coli* or *Pseudomonas aeruginosa* (Fig. S11). The *glcDF* genes we discovered fell into two clades, one that clustered together with the previously characterized *glcD* and *glcF* genes with high bootstrap support, and another (that included the EZ55 gene) that represented a separate evolutionary lineage intermediate between *glcD2* and the canonical *E. coli* three-protein enzyme.

The EZ55 genome also contained two genes similar to the *E. coli* lactate oxidase *lctD*. Bacterial lactate oxidases (LOX) have been shown to also operate on glycolate and are thought to be the ancestors of the H_2_O_2_-producing glycolate oxidase (GOX) enzyme found in plants [36]. Further analysis revealed that both the EZ55 and *E. coli* proteins aligned well with eukaryotic GOX as well as an H_2_O_2_-producing LOX from *Streptococcus pyogenes* [37] (Fig. S12). GOX/LOX candidates could not be found in any *Synechococcus* genome examined (including the two studied here), nor in any high-light clade *Prochlorococcus* genome (although two low-light strains, MIT9211 and NATL2A, did have candidate genes). It is thus possible that EZ55 can use the more efficient plant-like GOX pathway for glycolate catabolism, producing H_2_O_2_ as a by-product. We found no evidence of the beta-hydroxyaspartate cycle pathway for glycolate utilization [38] in any of the four genomes.

**Fig. 4:**
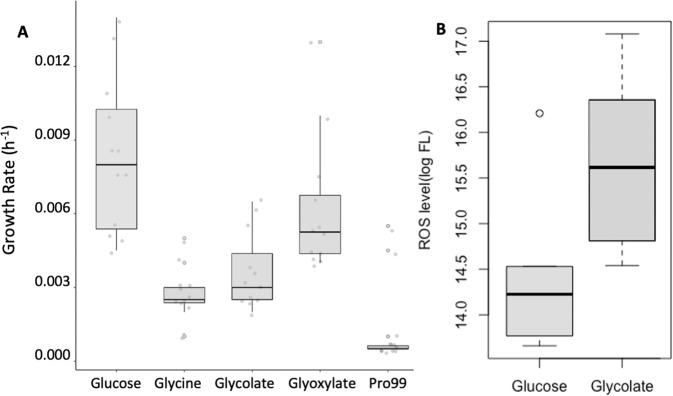
*Alteromonas* EZ55 utilization of photorespiratory metabolites. **A** Exponential growth rate of EZ55 on three photorespiration-related compounds, compared to a positive (glucose) and negative (unamended C-free Pro99 medium). **B** Intracellular H_2_O_2_ content in EZ55 cells growing on glucose or glycolate, represented as the average log fluorescence (FL) of DCFH-DA stained cells analyzed by flow cytometry.

Based on this genomic evidence, we concluded that both *Synechococcus* strains and EZ55 were capable of fully recovering 2-phosphoglycolate (2PG), the proximal product of photorespiration, using either the GCS, the TCA glyoxylate shunt, or glycerate (Fig. 3) [35]. At least one of these pathways was upregulated for EZ55 in co-culture with each cyanobacterial partner at 400 ppm pCO_2_ (Fig. 3). MIT9312, on the other hand, did not have a complete set of enzymes for converting 2PG to glyoxylate, and therefore we predicted that MIT9312 must excrete 2PG into the medium at a relatively constant rate during carbon fixation. We therefore investigated EZ55’s ability to grow on various metabolites related to 2PG. Axenic EZ55 cultures grew on glycolate, glyoxylate, and glycine as sole carbon sources (Fig. 4A), albeit with significantly lower growth rates than on glucose (ANOVA followed by Tukey post-hoc test, *p* < 0.05). We also measured intracellular H_2_O_2_ in EZ55 cultures growing on either glucose or glycolate (Fig. 4B). Glycolate-grown cultures had significantly higher H_2_O_2_-induced fluorescence than glucose cultures (ANOVA, *p* < 0.05), consistent with GOX activity in EZ55.

## Discussion

We aimed to understand the impact of changing pCO_2_ (400 vs. 800 ppm, representing current and projected year 2100 concentrations) on *Prochlorococcus* and *Synechococcus* and its effects on their interactions with the co-cultured heterotrophic “helper” bacterium *Alteromonas* sp. EZ55. Consistent with our previous research [7], EZ55 was more strongly affected by year 2100 pCO_2_ than any of the photoautotrophs in our study despite the primary dependence of the latter organisms’ metabolism on CO_2_. Strikingly, elevated pCO_2_ tended to reduce or eliminate the effect of co-culture on EZ55, with far fewer genes being significantly differentially transcribed relative to axenic EZ55 at the same pCO_2_. Thus, pCO_2_ strongly impacted the metabolic conversation between cyanobacteria and EZ55. Our detailed analysis of differentially regulated metabolic pathways suggested three mutually reinforcing mechanisms underlying this dynamic interaction: (i) pCO_2_ impacts on the release of ‘leaky’ cyanobacteria-derived metabolites, (ii) alteration of the dynamics of competition over inorganic nutrients between the co-cultured organisms, and (iii) modulation of bacterial and phytoplankton stress states. We explore each of these mechanisms in further detail below.

### Carbon cycling of “leaky” metabolites in co-culture

**Fig. 5:**
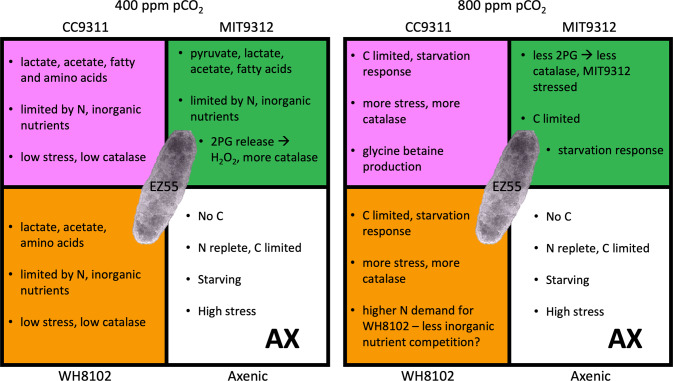
Proposed reconstruction of *Alteromonas* EZ55 ecophysiology. Reconstructions are shown for four different community contexts (axenic culture, or co-culture with *Prochlorococcus* MIT9312, *Synechococcus* WH8102, or *Synechococcus* CC9311) at 400 or 800 ppm pCO_2_, reflecting possible changes in the availability of C compounds, growth limiting factors, and stress conditions consistent with differential gene transcription observations. EZ55 image was obtained by cryoelectron microscopy from the sessions reported in Hennon et al. [7]. Background colors for each partner correspond to the bar colors in Fig. 3.

The media we used for coculturing phytoplankton and bacteria contained no exogenous carbon sources; therefore, EZ55 was dependent on cyanobacterial exudates to grow, and it is likely that much of its changed transcription reflected changing availability of extracellular metabolites in the medium. The significant upregulation of carbon catabolism and transport genes as well as chemotaxis genes in co-cultures relative to axenic EZ55 supports the view that bacterial remineralization of cyanobacteria-secreted organic compounds is a driving force in these simple ecosystems. Additionally, changes in transcription of carbohydrate catabolism and transport genes provide clues as to which metabolites were being secreted under different experimental conditions (Fig. 5).

Like all oxygenic phototrophs, the cyanobacteria studied here fix carbon using the enzyme rubisco, which also catalyzes the undesirable photorespiration reaction leading to the production of 2PG instead of photosynthate. Phytoplankton in the field and in culture have been observed to excrete low molecular weight carboxylic acids including glycolate [39–41]. Photorespiratory glycolate is one of the most abundant sources of carbon in the oceans [38] and a preferred growth substrate for some marine heterotrophic bacteria [42]. Moreover the bacterial *glcD* gene for converting glycolate to glyoxylate is ubiquitously transcribed in the ocean [41, 43]. Although EZ55 lacks a specific transporter for glycolate, it can be taken up by the cell using the same transporters used for acetate and lactate uptake [44, 45], both of which were upregulated in co-culture conditions at 400 ppm (Fig. 3). Our data also showed differential regulation of enzymes involved in glycolate catabolism pathways, with at least one pathway upregulated in co-culture with each cyanobacterial strain (Fig. 3). We further demonstrated that EZ55 cultures were capable of growth on glycolate as a sole source of carbon, possibly using a novel GlcDF fusion protein (Fig. S11) and/or a plant-like LOX/GOX enzyme (Fig. 4). Thus, photorespiratory byproducts are likely a source of carbon for EZ55 in these cultures, particularly in the presence of MIT9312, which has no detectable enzymes for reclaiming 2PG on its own.

There was also evidence that EZ55 utilized amino acids, organic acids, and fatty acids produced by phytoplankton under certain conditions in these cultures (Fig. S9). Lactate, acetate, and propanoate transporters and catabolism pathways were upregulated in co-culture with all cyanobacteria, as was pyruvate dehydrogenase with MIT9312, but only at 400 ppm. Both valine and glycine catabolism were also upregulated at 400 ppm in co-culture with the two *Synechococcus* strains, and fatty acid catabolism was upregulated in co-culture with MIT9312 and CC9311 at 400 ppm pCO_2_. Most of these substances have been directly or indirectly observed in cyanobacterial cultures in previous studies. For example, glycolate, lactate, acetate, and pyruvate have been directly measured in *Prochlorococcus* spent media [39], and co-culture with *Prochlorococcus* can fulfill the SAR11 growth requirement for glycine and pyruvate [46]. Fatty acid catabolism genes may have targeted membrane vesicles which are abundantly released by *Prochlorococcus* and other marine bacteria and may be a significant source of carbon for heterotrophs in the ocean [47, 48]; if so, future studies should investigate if WH8102 produces fewer vesicles than the other two cyanobacteria, explaining the differential transcription of beta-oxidation genes observed here.

Valine, fatty acid, and propanoate catabolic pathways intersect with the formation of propanoyl-coA which in bacteria is generally fed into the TCA cycle through the methylcitrate pathway [49], which was significantly downregulated at 400 ppm in co-culture with all cyanobacteria even though other genes in these pathways were upregulated. Therefore, it is not clear what the ultimate fate of carbon from these sources is, although it is possible that EZ55 may be able to convert propanoyl-coA into a TCA cycle intermediate through another alternative pathway (e.g. as has been described in *Mycobacterium tuberculosis* via the methylmalonyl pathway [50]).

Notably, gene transcription related to the utilization of all these products declined at 800 ppm pCO_2_ (Figs. 3, S8, S9). This was not unexpected for enzymes in the glycolate utilization pathways, as the increased CO_2_/O_2_ ratio at 800 ppm should decrease the rate of photorespiration relative to carbon fixation and therefore the availability of photorespiratory metabolites like glycolate [51, 52]. It is not clear, however, why organic and fatty acids would be less abundant in cyanobacterial exudates at 800 ppm. One possibility is that cyanobacteria release fewer of these compounds into the medium at high pCO_2_ because of a change in their internal redox state under these conditions favoring full oxidation of photosynthate. If future pCO_2_ conditions fundamentally alter the character of phytoplankton exudates, this could have profound implications for evolution and ecosystem functioning in future oceans.

### Evidence for inorganic nutrient limitation and competition

Autotrophic cyanobacteria and heterotrophic EZ55 were unlikely to compete over carbon under our experimental conditions, but we observed evidence of competition over inorganic nutrients such as N, P, and Fe. EZ55 phosphate, ammonium, and iron transporters, nitrogen regulatory protein P-II, and glutamine synthetase (the primary gateway for N assimilation in bacteria) were all more highly transcribed for all co-cultures compared to axenic cultures at 400 ppm pCO_2_ (Fig. S6), suggesting a switch from axenic carbon limitation to nutrient limitation in the presence of a continual supply of photosynthetically derived carbon (Fig. 5). On the other hand, few nutrient transporters were upregulated compared to axenic under 800 ppm pCO_2_. Although gene transcription data alone is not sufficient to conclude whether *Alteromonas* is limited by inorganic or organic nutrients, the reduced importance of nutrient acquisition suggests that EZ55 is carbon limited under these conditions just as it is in the absence of cyanobacteria.

There were comparatively few species-specific changes in EZ55 nutrient transporter gene transcription. One example was an ammonium transporter, which was strongly upregulated in co-culture with both open ocean cyanobacteria (MIT9312 and WH8102) at 400 ppm pCO_2_. This may reflect a response to a comparatively high affinity for N in cyanobacteria adapted to the permanently oligotrophic open ocean, making them much stronger competitors for limiting N than coastal CC9311. N competition with EZ55 has been observed to increase the relative competitive fitness of *Prochlorococcus* vs. *Synechococcus* (coastal strain WH7803) in 3-way co-cultures [53]. In contrast, WH8102 appears to have higher N demand under 800 ppm pCO_2_, significantly upregulating a nitrate transporter and several genes related to urea utilization (Fig. S2). This may be explained by the enhanced transcription of carbon fixation genes and faster exponential growth rates observed in WH8102 at elevated pCO_2_, increasing N demand, and may indicate that WH8102 was C limited at 400 ppm.

It is important to note that different N sources were provided in PEv medium (in which axenic EZ55 and MIT9312 co-cultures were grown) and SEv medium (in which CC9311 and WH8102 co-cultures were grown), with NH_4_^+^ in the former and NO_3_^-^ in the latter. However, we do not think this difference can explain the observed changes in gene regulation, since EZ55 is capable of growth using either N source. It is interesting to note, however, that EZ55’s ammonium transporter was upregulated in both media types (Fig. S6), suggesting it may be benefitting from ammonium excreted by *Synechococcus* in SEv co-cultures.

### Impacts of co-culture and pCO_2_ on stress conditions

EZ55 showed less transcription of stress-related genes at 400 than 800 ppm pCO_2_, and also less evidence of stress in co-culture with any cyanobacterium than in axenic culture by itself. Nearly every gene in the EZ55 genome related to protection from H_2_O_2_ was downregulated in co-culture at 400 ppm, as were a suite of other stress-related genes (Fig. 2); on the other hand, many of these genes were significantly upregulated relative to axenic conditions at 800 ppm. Additionally, at 800 ppm there was a pronounced difference in EZ55 H_2_O_2_ defense gene transcription between cyanobacterial partners. As we described previously [7], both monofunctional catalases were downregulated at 800 ppm in co-culture with MIT9312, as were 2 of 3 alkylhydroperoxide reductase genes (although the third was significantly upregulated). In contrast, the monofunctional catalase genes were significantly upregulated in co-culture with WH8102 at 800 ppm. Elevated transcription of genes involved in the biosynthesis of glycine betaine, an osmoprotectant which has also been shown to function as an antioxidant [54, 55], provides further evidence for increased oxidative stress in co-culture with *Synechococcus* at 800 ppm in EZ55.

Some indication of the mechanism behind EZ55’s changing stress level under co-culture and elevated pCO_2_ can be seen in the dynamics of three stress-related RNA polymerase sigma factors. Both *rpoE* and *rpoH*, responsible for controlling envelope and heat stress regulons, respectively, were downregulated at 400 ppm in co-culture relative to axenic and 800 ppm conditions; *rpoE* was significantly upregulated at 800 ppm pCO_2_. These trends are consistent with starvation-induced oxidative stress under both axenic and 800 ppm conditions, as discussed above. In contrast, *rpoS* was upregulated at 400 ppm pCO_2_, strongly so in co-culture with MIT9312. RpoS is a specialized sigma factor that accumulates under conditions of nutrient deprivation or as cells enter the stationary phase and serves to increase general stress resistance [56, 57]. For example, in *Escherichia coli* RpoS was shown to play a crucial role for survival during nitrogen deprivation [58]. While the decoupling of the transcription of oxidative stress genes like catalase from *rpoS* transcription was unexpected, *rpoS* trends are consistent with EZ55 being nutrient limited at 400 ppm pCO_2_ (Fig. S6) and with the upregulation of catalase in co-culture with MIT9312, but not WH8102 or CC9311, at 400 ppm (Fig. 2).

In contrast to EZ55, differentially transcribed genes related to stress responses were rare in cyanobacteria at 800 ppm. While both MIT9312 and WH8102 had significant growth impairments at 800 ppm (Fig. S1), there was little evidence of a stress-specific gene transcription response in either strain. DNA mismatch repair genes were enriched as a group at 800 ppm in *Prochlorococcus*, although the only individual stress-related protein that was differentially transcribed was a HLI protein that was strongly downregulated at 800 ppm. No stress-related genes or gene sets were enriched in WH8102, and the small number of differentially transcribed stress genes in CC9311 (e.g., heat-shock and HLI proteins) were all downregulated at 800 ppm. This could indicate a dependence of both MIT9312 and WH8102 on their co-cultured EZ55 partner for protection, as neither of these cyanobacterial genomes contains catalase or several other stress-response genes common in heterotrophic bacteria. It could also indicate that they have different stress response mechanisms than those that have been characterized in heterotrophic bacteria; for instance, several hypothetical proteins of unknown function were differentially regulated in each cyanobacterium between the pCO_2_ conditions. Finally, it is possible that the stresses experienced by MIT9312 and WH8102 occurred in the initial days after transfer into fresh media (i.e., the significantly extended lag period observed for both), and were alleviated by the late log phase when the cultures were sampled for RNA sequencing.

### Summary overview of metabolic responses

We have shown that the response to elevated pCO_2_ in our algal:bacterial co-cultures was driven more by interspecies interactions than by CO_2_-specific responses themselves. While it is important to note that we do not have direct culture-based evidence for some of these claims, we feel that gene transcription evidence is strong for several conclusions regarding the interactions in our cultures (Fig. 5).

First, increased pCO_2_ appears to have fundamentally altered the amount and/or types of carbon compounds secreted by all three cyanobacterial strains examined, placing EZ55 into a stationary-phase metabolic state nearly indistinguishable to being in culture media with no added carbon source at all. We suggest that this is driven directly by the higher CO_2_:O_2_ ratio, which lowered the rate of photorespiration and subsequent release of 2PG and/or glycolate and indirectly may have reduced the amount of incompletely oxidized carbon released by cyanobacteria by changing the intracellular redox state [59]. Possibly because of the changing supply of carbon, EZ55 also appeared to transition away from a state of nutrient competition with its cyanobacterial partners, exemplified by decreased transcription of nutrient transporters at elevated pCO_2_ (Fig. S6).

Second, co-culture at 400 ppm clearly reduced stress on EZ55 relative to either axenic growth or co-culture growth at 800 ppm, possibly due to the provision of a more reliable source of C as described above by the cyanobacterial partner under these conditions. In contrast, both MIT9312 and WH8102 clearly experienced elevated stress, potentially related to the changes in EZ55’s metabolism under these conditions. One of the major conclusions from our previous work [7] was the finding that EZ55 reduced catalase transcription at 800 ppm pCO_2_, eliminating the “helper” effect that *Prochlorococcus* depends on to grow in culture [13, 14]. In this work we see that the catalase response in co-culture with MIT9312 was opposite that in co-culture with the two *Synechococcus* strains. One possible explanation for this lies in the fact that MIT9312, unlike the other three strains in this study, did not possess a complete 2PG catabolism pathway and therefore likely excreted this product where it was subsequently catabolized by EZ55. We confirmed by genomic analysis (Figs. S10–S13) and culture experiments (Fig. 4) that EZ55 was able to grow on glycolate as a sole carbon source, and that its intracellular H_2_O_2_ concentration was elevated compared to growth on glucose. We suggest that more 2PG was secreted by MIT9312 at 400 ppm pCO_2_ due to the lower CO_2_:O_2_ ratio, and that growth on this carbon source increased EZ55’s internal oxidative stress load, resulting in higher transcription of H_2_O_2_ defenses such as catalase (Fig. 2). If true, this provides one possible explanation of why the “helper” relationship broke down at elevated pCO_2_ – by leaking 2PG as a readily available growth substrate for EZ55 at 400 ppm, MIT9312 forced EZ55 to maintain a high degree of intracellular ROS defense, leading to the well-characterized ability of EZ55 to cross-protect *Prochlorococcus* strains from the relatively lower H_2_O_2_ concentrations in the bulk environment, and allowing MIT9312 to eliminate two energetically costly enzymatic pathways. When higher pCO_2_ reduced the rate of photorespiration, EZ55’s need to produce excess catalase decreased, resulting in lower levels of protection, and concomitant growth impairments, for MIT9312.

This is an example of how leaky Black Queen functions allow organisms like *Prochlorococcus* to streamline their metabolism while simultaneously creating stable interdependencies within their communities. However, it also shows how Black Queen-stabilized exchanges can break down. If our hypothesized relationship between pCO_2_ and catalase production is correct, then this system depends on the passive release of a metabolic by-product that evolved under a set of atmospheric pCO_2_ conditions that have been largely stable for thousands of years – but this leaves the system particularly vulnerable to the rapid changes in pCO_2_ currently taking place and may leave *Prochlorococcus* with no protection at all in the future ocean. If *Prochlorococcus* is outcompeted by less-streamlined competitors, this could reduce the overall efficiency of primary production in the open ocean gyres with possible positive feedbacks on CO_2_ accumulation in the atmosphere. Subsequent experiments should examine whether *Prochlorococcus* can overcome this imbalance through adaptive evolution quickly enough to avoid serious disruptions of its current niche.

In conclusion, these results provide further support for the observation that axenic cultures do not provide a good window into the behavior of natural communities. The metabolism of *Alteromonas* sp. EZ55, a ubiquitous consumer in the ocean, was strongly dependent on its community context, and relatively subtle shifts in the chemical environment induced by elevated pCO_2_ were sufficient to significantly remodel its physiology. Moreover, the transcriptional response of EZ55 to changing pCO_2_ was much greater than that of any of the photoautotrophs examined, suggesting that more work is needed to understand the often-ignored heterotrophic bacteria associated with marine primary producers and how they will respond to global ocean change. Thus, further research is indicated on some of our core findings and hypotheses (e.g., the role of 2PG, and the nature of the carbon exchanged between the cyanobacteria and *Alteromonas*) via metabolomics or direct substrate measurements. These results further highlight the importance of laboratory experiments using co-cultures as an experimentally tractable intermediate between oversimplified axenic cultures and overly complicated natural communities. They also highlight the dominant role that primary producers play in determining the metabolism and interactions of the organisms that depend on them for sustenance.

## Supplementary Information

Supplemental Information

Table S1

Table S2

Table S3

Table S4

Table S5

Table S6

Table S7

Table S8

Table S9

Table S10

Table S11

Table S12

Table S13

Table S14

## Data Availability

Our sequences files are accessible from the National Center for Biotechnology Information (BioProject PRJNA377729, Sequence Read Archive accession numbers SRX2619948-SRX2619957, SRX3033334-SRX3033345, and SRX14411251-SRX14411274). All data generated or analyzed from our sequence files as well as the raw data from our culture-based experiments and phylogenetic analyses is archived at bco-dmo.org as datasets 882409, 882390, and 881942.
