# Supplementary material for: Community context and pCO2 impact the transcriptome of the “helper” bacterium Alteromonas in co-culture with picocyanobacteria
Source: ISME Commun. 2022 Nov 15;2:113. doi: 10.1038/s43705-022-00197-2 (PMC9723591; doi:10.1038/s43705-022-00197-2)
Supplement: Supplementary file 1 — Supplemental Information [file 43705_2022_197_MOESM1_ESM.pdf]

Supplementary Information for

Community context and pCO_2 remodel the transcriptome of the "helper" bacterium *Alteromonas* in co-culture with picocyanobacteria

Marcelo Malisano Barreto Filho[1], Zhiying Lu[1], Melissa Walker[1], and J. Jeffrey Morris[1,#]

[1] University of Alabama at Birmingham Department of Biology, 1300 University Blvd CH464, Birmingham AL 35294 USA

[#] Author for correspondence: evolve@uab.edu

This document includes:

Legends for Supplemental tables

Supplemental Figs. S1 to S12

Supplemental Tables (all are .csv files available with the supplemental material)

Table S1. Differentially regulated genes between pCO_2 conditions for *Prochlorococcus* MIT9312, *Synechococcus* CC9311, and *Synechococcus* WH8102

Table S2. Differentially regulated genes in *Alteromonas* EZ55 between pCO_2 conditions

Table S3. Differentially regulated genes in *Alteromonas* EZ55 between coculture conditions

Table S4. Differentially regulated genes in *Alteromonas* EZ55 between pCO_2 conditions that are affected by co-culture condition (i.e., the interaction between pCO_2 and coculture)

Table S5. Differentially regulated stress-related genes in *Alteromonas* EZ55 in response to the general response to coculture, and coculture with specific cyanobacteria at either ambient (400 ppm) or elevated (800 ppm) pCO_2.

Table S6. Differentially regulated Transporter-related genes in *Alteromonas* EZ55 in response to the general response to coculture, and coculture with specific cyanobacteria at either ambient (400 ppm) or elevated (800 ppm) pCO_2.

Table S7. Differentially regulated Chemotaxis-related genes in *Alteromonas* EZ55 in response to the general response to coculture, and coculture with specific cyanobacteria at either ambient (400 ppm) or elevated (800 ppm) pCO_2.

Table S8. Differentially regulated Metabolism-related genes in *Alteromonas* EZ55 in response to the general response to coculture, and coculture with specific cyanobacteria at either ambient (400 ppm) or elevated (800 ppm) pCO_2.

Table S9. Differentially regulated genes in *Alteromonas* EZ55 in response to coculture with *Prochlorococcus* sp. MIT9312 at 400 ppm pCO_2

Table S10. Differentially regulated genes in *Alteromonas* EZ55 in response to coculture with *Synechococcus* sp. CC9311 at 400 ppm pCO_2

Table S11. Differentially regulated genes in *Alteromonas* EZ55 in response to coculture with *Synechococcus* sp. WH8102 at 400 ppm pCO_2

Table S12. Differentially regulated genes in *Alteromonas* EZ55 in response to coculture with *Prochlorococcus* sp. MIT9312 at 800 ppm pCO_2

Table S13. Differentially regulated genes in *Alteromonas* EZ55 in response to coculture with *Synechococcus* sp. CC9311 at 800 ppm pCO_2

Table S14. Differentially regulated genes in *Alteromonas* EZ55 in response to coculture with *Synechococcus* sp. WH8102 at 800 ppm pCO_2

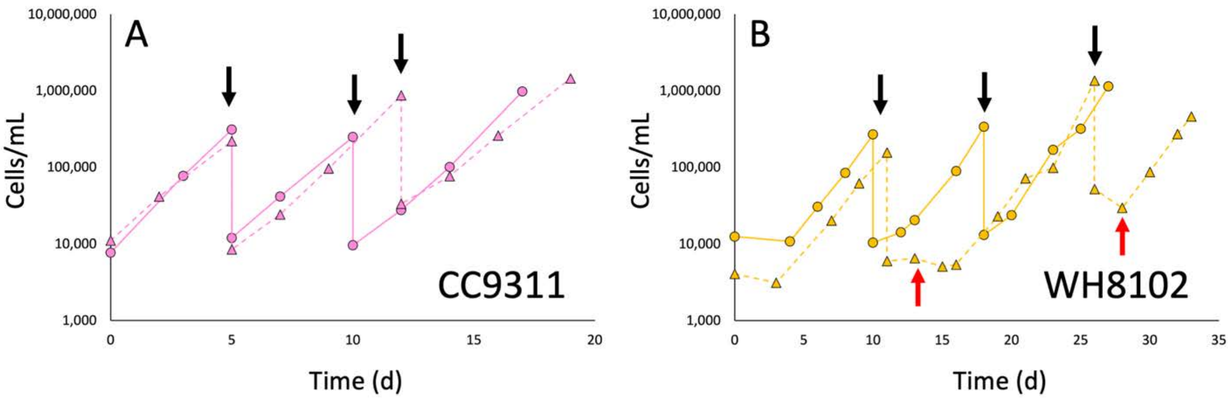

Figure S1. Growth dynamics of *Synechococcus* CC9311 (A) or *Synechococcus* WH8102 (B) under 400 (solid line) or 800 (dashed line) ppm pCO_2 conditions. Each graph shows a representative culture series across three subsequent transfer cycles; cultures were transferred into fresh media by 26-fold dilution whenever they crossed 2.6×10^5 cells mL^{-1}. Transfers were performed at the points indicated by black arrows. The red arrows in (B) shows an extended lag period (left arrow) and a moderate cell die-off (right arrow) illustrative of the growth problems experienced by WH8102 at 800 ppm pCO_2.

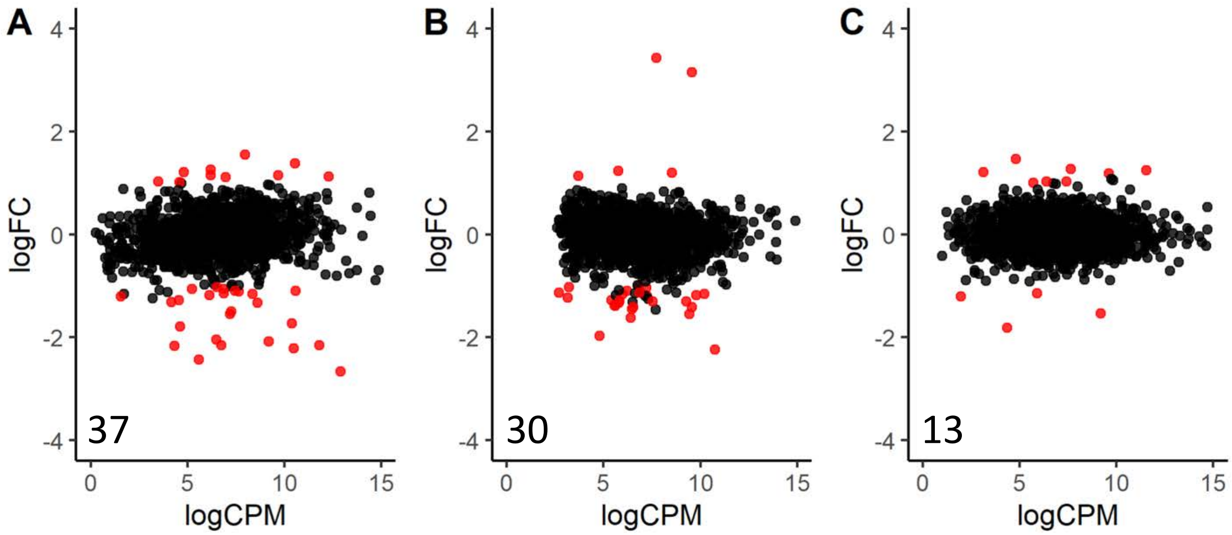

Figure S2. Differential gene transcription in *Prochlorococcus* MIT9312 (A), *Synechococcus* WH8102 (B) and *Synechococcus* CC9311 (C) between growth at 400 and 800 ppm pCO_2. Transcription for each gene is shown on the x-axis as the average abundance in log_2 counts per million (CPM), and the log_2 of the fold change (FC) on the y-axis. Genes which are significantly differentially transcribed ($p < 0.05$ and $|log_2(FC)| > 1$) are highlighted in red. Values in the bottom left corner of each plot indicate the number of genes significantly differentially regulated in each case.

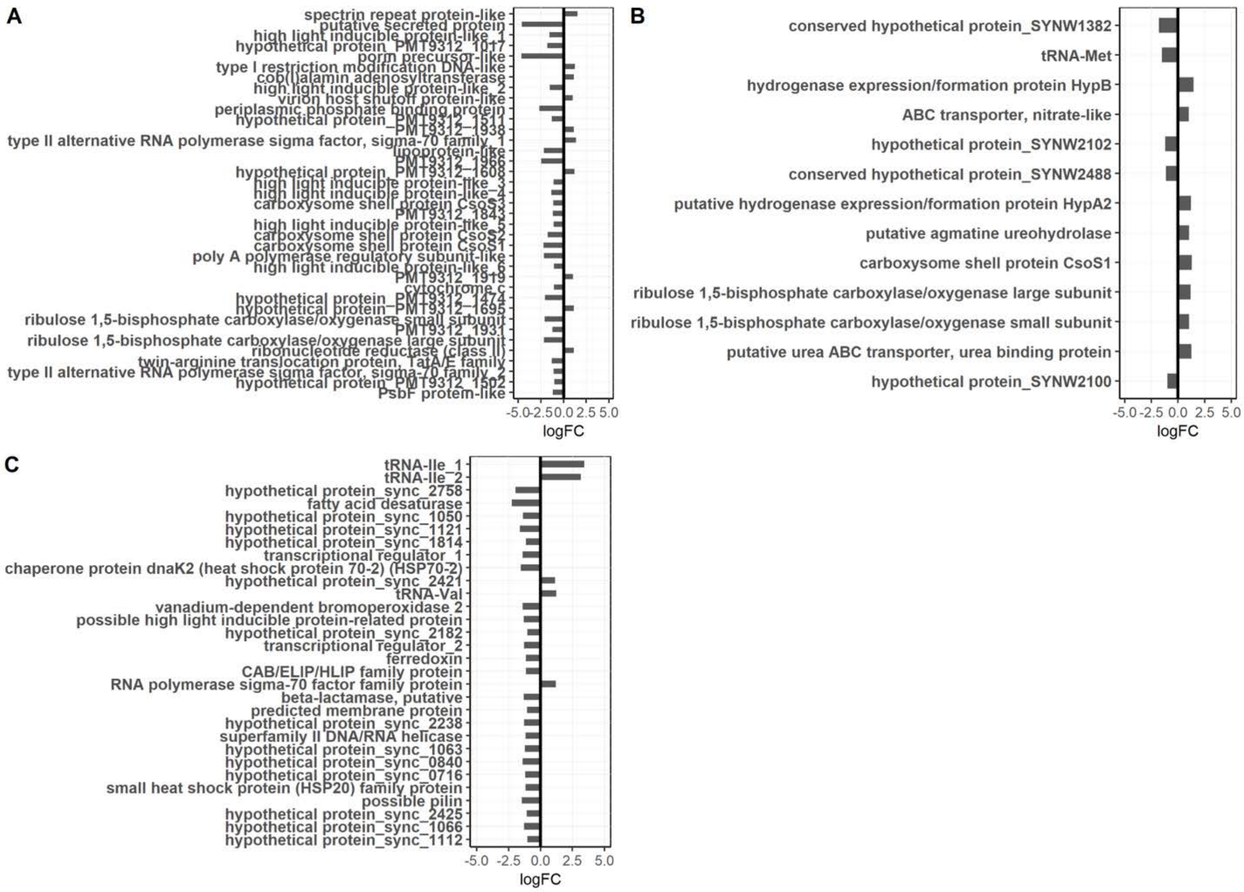

Figure S3. Differentially transcribed individual gene products in *Prochlorococcus* MIT9312 (A), *Synechococcus* WH8102 (B) and *Synechococcus* CC9311 (C) between growth at 400 and 800 ppm pCO_2. Positive or negative transcribed for each gene product is shown on the x-axis as the log_2 of the fold change (FC).

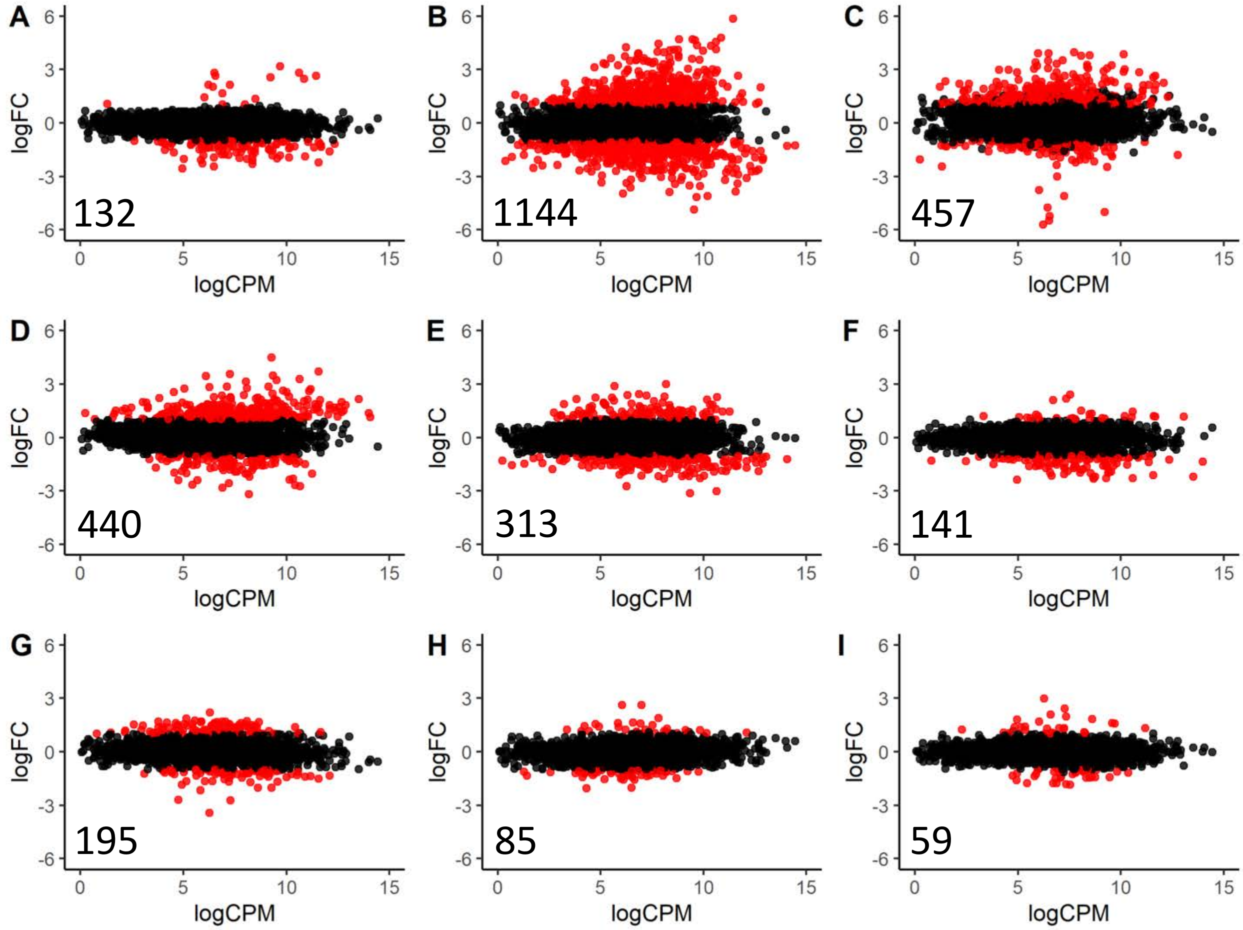

Figure S4. Differential gene transcribed in *Alteromonas* EZ55 under various experimental conditions. Transcription for each gene is shown on the x-axis as the average abundance in $\log_2$ counts per million (CPM) and the $\log_2$ of the fold change (FC) on the y-axis. Genes which are significantly differentially transcribed ($p < 0.05$ and $|\log_2(FC)| > 1$) are highlighted in red. Values in the bottom left corner of each plot indicate the number of genes significantly differentially regulated in each case. (A) General response to 800 ppm pCO2; (B) general response to co-culture with cyanobacteria; (C) genes that are differentially transcribed between pCO_2 conditions in one co-culture context but not the other; (D-F) genes that are differentially transcribed only in coculture with MIT9312, CC9311, or WH8102, respectively, at 400 ppm pCO_2; (G-I) genes that are differentially transcribed only in coculture with MIT9312, CC9311, or WH8102, respectively, at 800 ppm pCO_2.

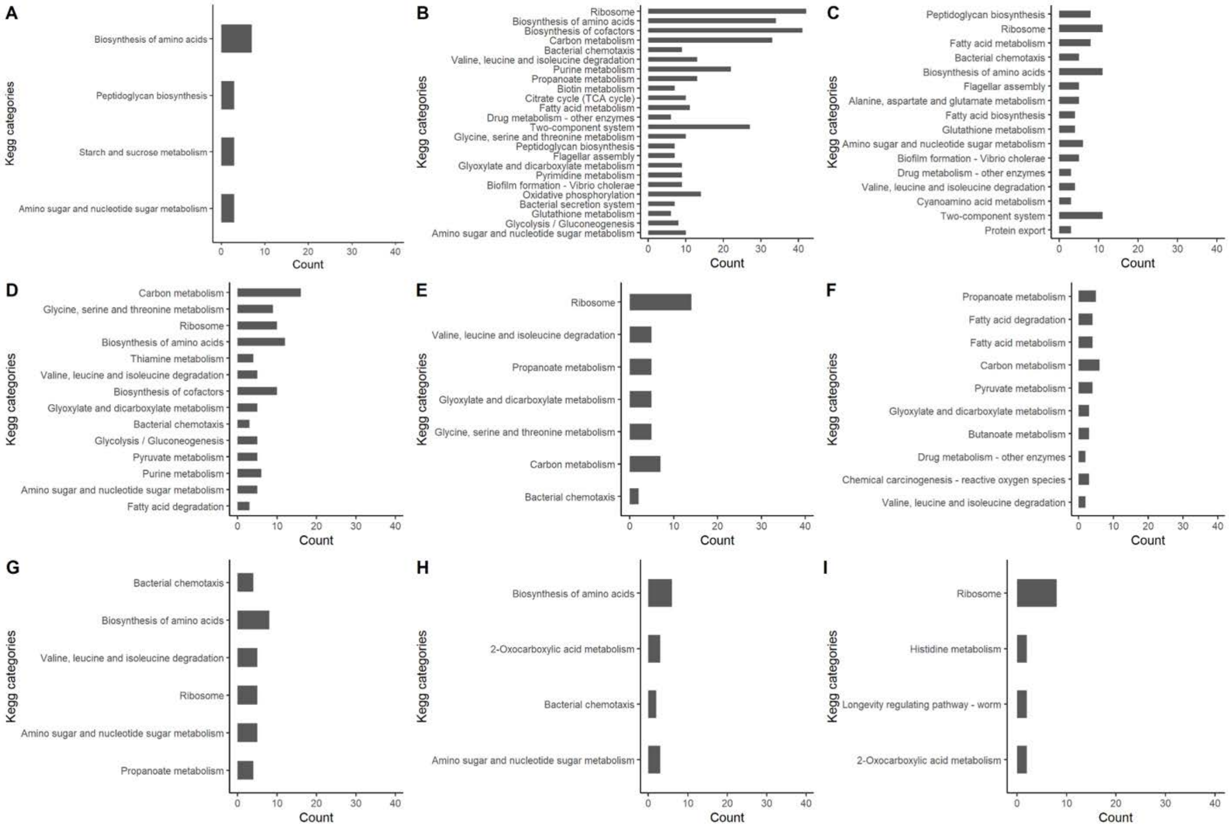

Figure S5. Plots for over-representation analysis (ORA) of significantly-changed KEGG pathways ($p < 0.01$) in *Alteromonas* EZ55 under different experimental conditions. ORA was implemented using hypergeometric tests according to Wu et al., (2021). (A) General response to 800 ppm pCO2; (B) general response to co-culture with cyanobacteria; (C) genes that are differentially transcribed between pCO_2 conditions in one co-culture context but not the other; (D-F) genes that are differentially transcribed only in coculture with MIT9312, CC9311, or WH8102, respectively, at 400 ppm pCO_2; (G-I) genes that are differentially transcribed only in coculture with MIT9312, CC9311, or WH8102, respectively, at 800 ppm pCO_2.

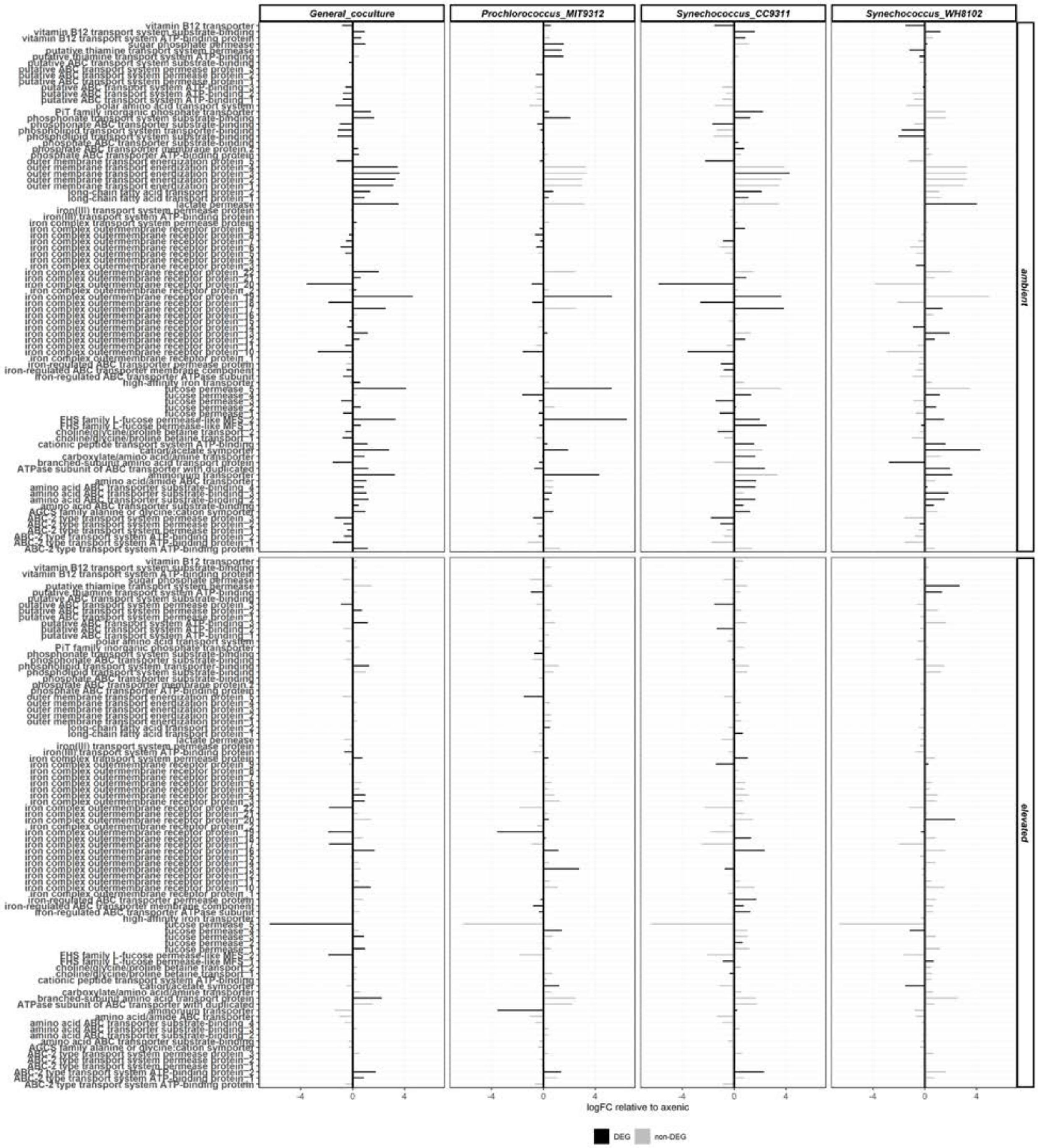

Figure S6. Transporter-related genes in EZ55 representing the general response to co-culture (column 1), and co-culture with specific cyanobacteria (columns 2-4) at 400 or 800 ppm pCO_2, relative to axenic conditions at the same pCO_2. Log_2 fold change (logFC) is plotted relative to axenic EZ55 under the same pCO_2 condition. DEG products are highlighted in black. Black bars in column 1 indicate the average co-culture response is significantly different from the axenic response at the same pCO_2; black bars in columns 2-4 indicate significant difference between the specific cyanobacterial response and the general coculture response shown in column 1.

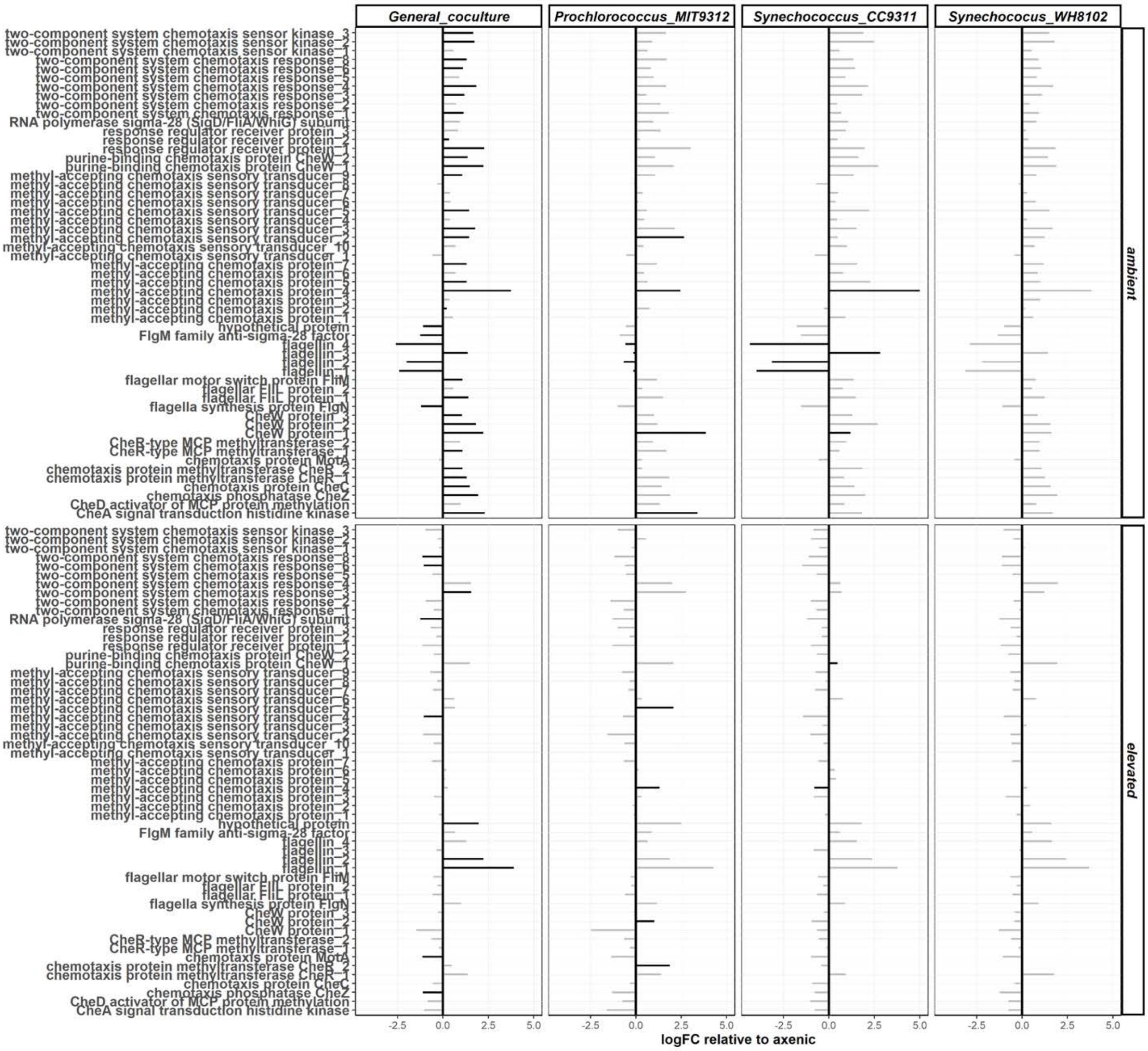

Figure S7. Summary plot of chemotaxis and two-component sensory system gene products in *Alteromonas* EZ55 representing the general response to co-culture (column 1), and co-culture with specific cyanobacteria (columns 2-4) at either 400 or 800 ppm pCO_2, relative to transcription under axenic conditions at the same pCO_2. Log_2 fold change (logFC) is plotted on the x-axis relative to axenic *Alteromonas* under the same pCO_2 condition. Differentially transcribed gene products ($p < 0.05$ and logFC > 1) are highlighted in black. Black bars in column 1 indicate the average co-culture response is significantly different from the axenic response at the same pCO_2; black bars in columns 2-4 indicate significant difference between the specific cyanobacterial response and the general coculture response shown in column 1.

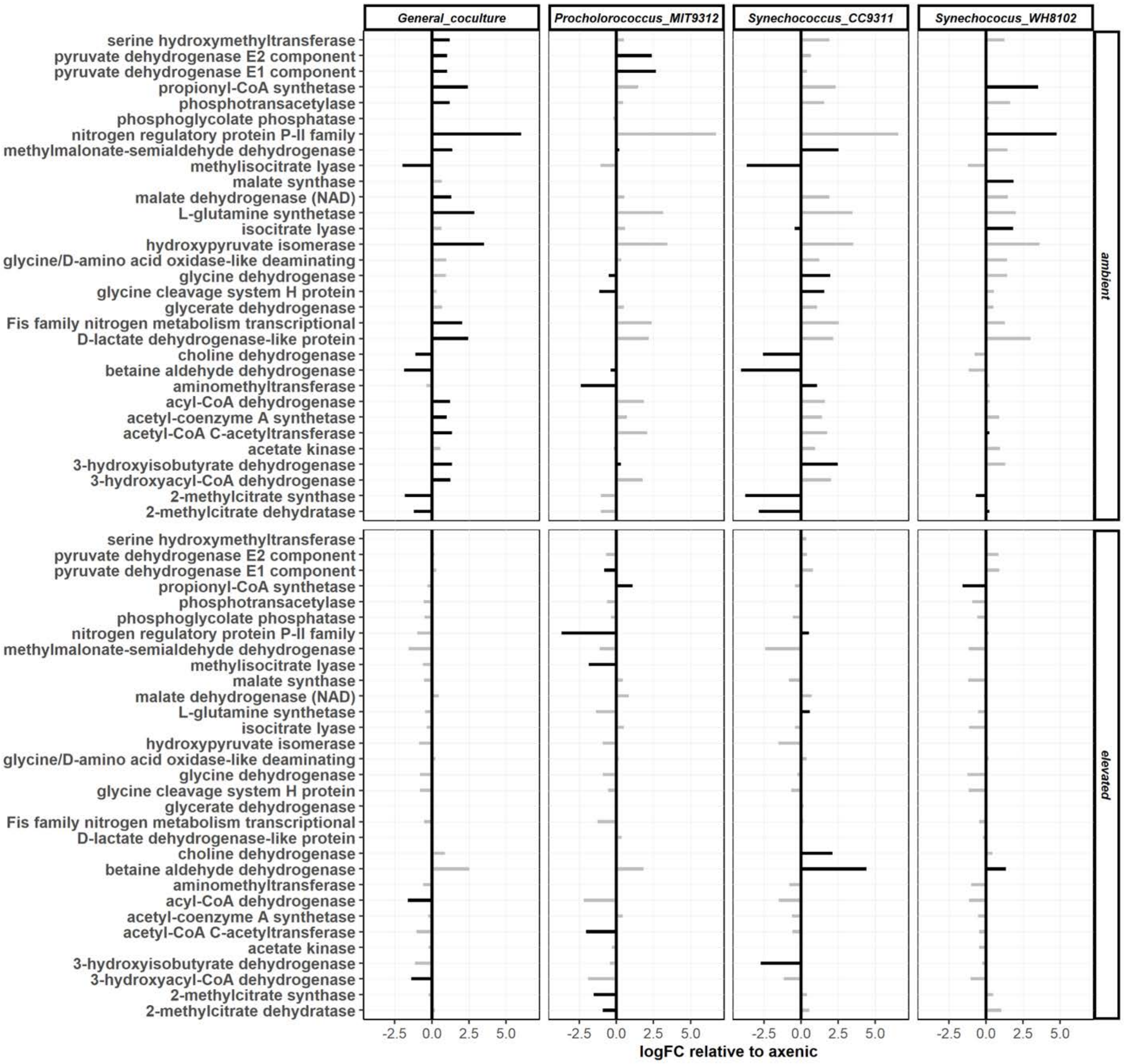

Figure S8. Summary plot of metabolism-related gene products in *Alteromonas* EZ55 representing the general response to co-culture (column 1), and co-culture with specific cyanobacteria (columns 2-4) at either 400 or 800 ppm pCO_2, relative to transcription under axenic conditions at the same pCO_2. Log_2 fold change (logFC) is plotted on the x-axis relative to axenic *Alteromonas* under the same pCO_2 condition. Differentially transcribed gene products ($p < 0.05$ and $logFC > 1$) are highlighted in black. Black bars in column 1 indicate the average co-culture response is significantly different from the axenic response at the same pCO_2; black bars in columns 2-4 indicate significant difference between the specific cyanobacterial response and the general coculture response shown in column 1.

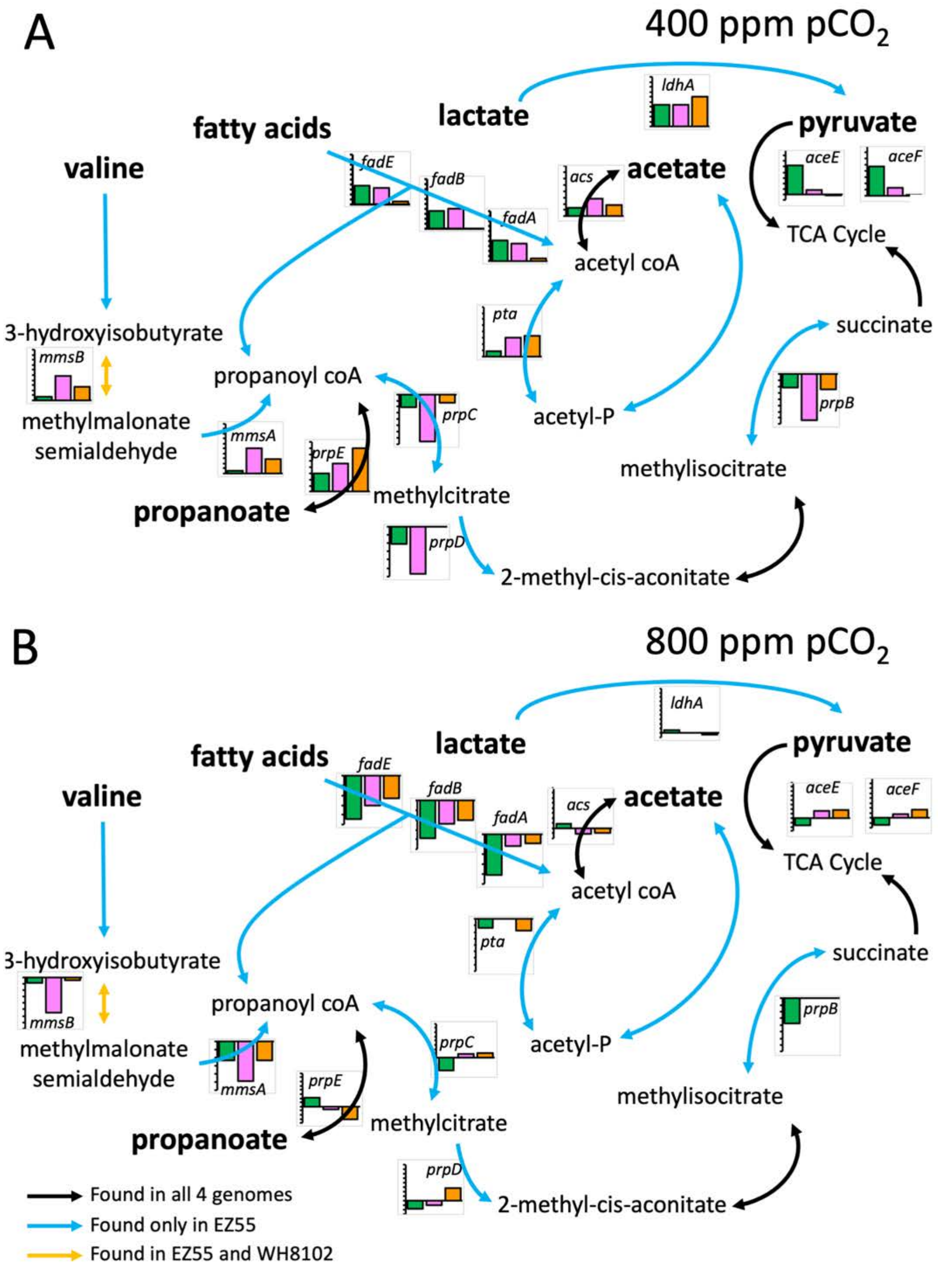

Figure S9. Reconstruction of metabolic pathways involving genes not directly involved in the recovery of photorespiratory byproducts that were significantly differentially regulated in *Alteromonas* EZ55 between axenic and co-culture conditions at 400 (A) or 800 (B) ppm pCO_2. Small inset graphs indicate the log fold change of transcription, relative to axenic culture, in co-culture with MIT9312 (green bars), CC9311 (pink bars), or WH8102 (orange bars). Gene names correspond to the related genes in *E. coli*. Only genes that are differently transcribed between axenic and co-culture in at least one condition are shown. Metabolites in bold print are hypothesized to be phytoplankton exudates used by EZ55 under certain conditions. Arrow colors indicate which of the four genomes are capable of performing the indicated reaction based on annotated gene functions; see inset legend in panel (B) for explanation.

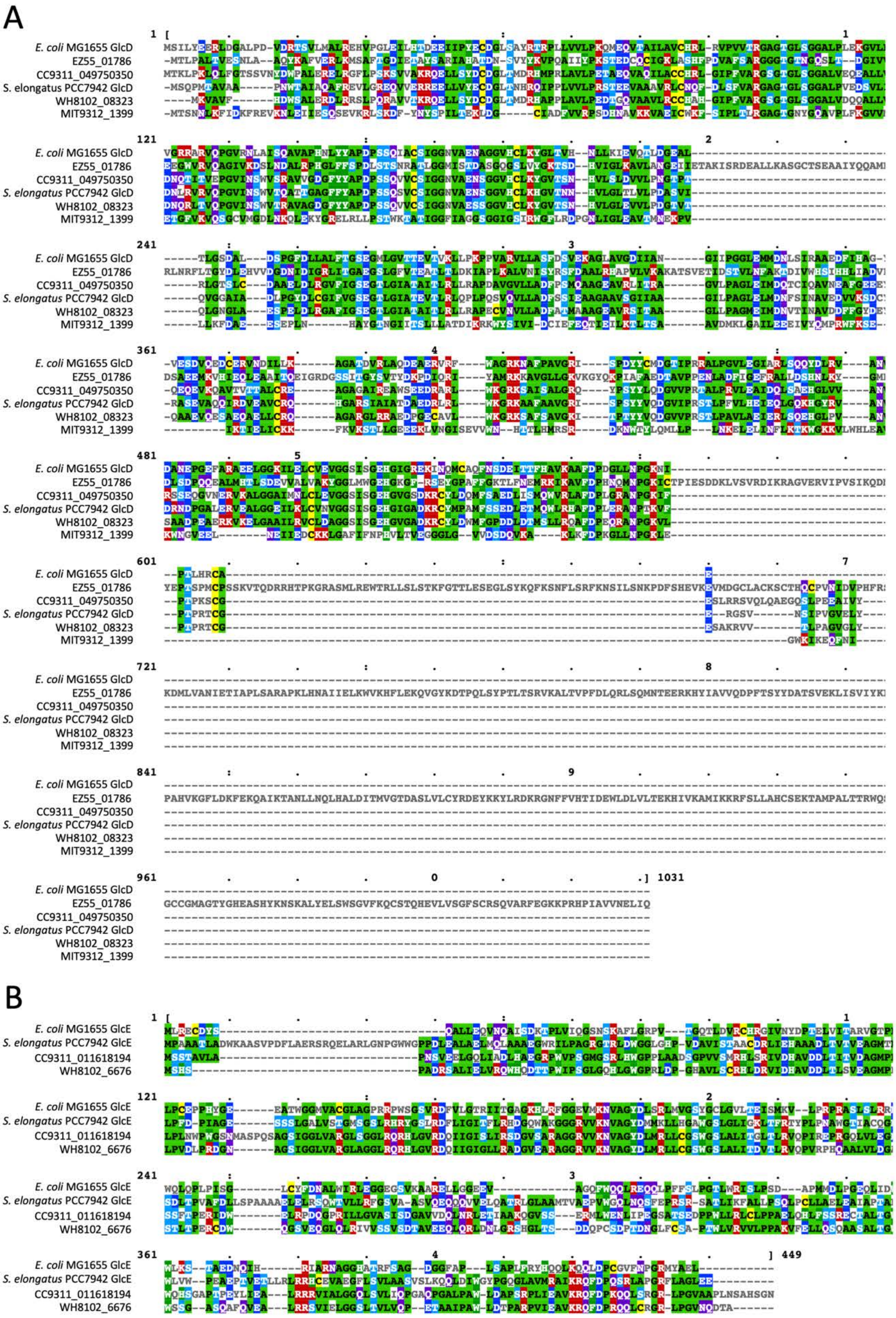
A
E. coli MG1655 GlcD
EZ55_01786
CC9311_049750350
S. elongatus PCC7942 GlcD
WH8102_08323
MIT9312_1399
B
E. coli MG1655 GlcE
S. elongatus PCC7942 GlcE
CC9311_011618194
WH8102_6676

C

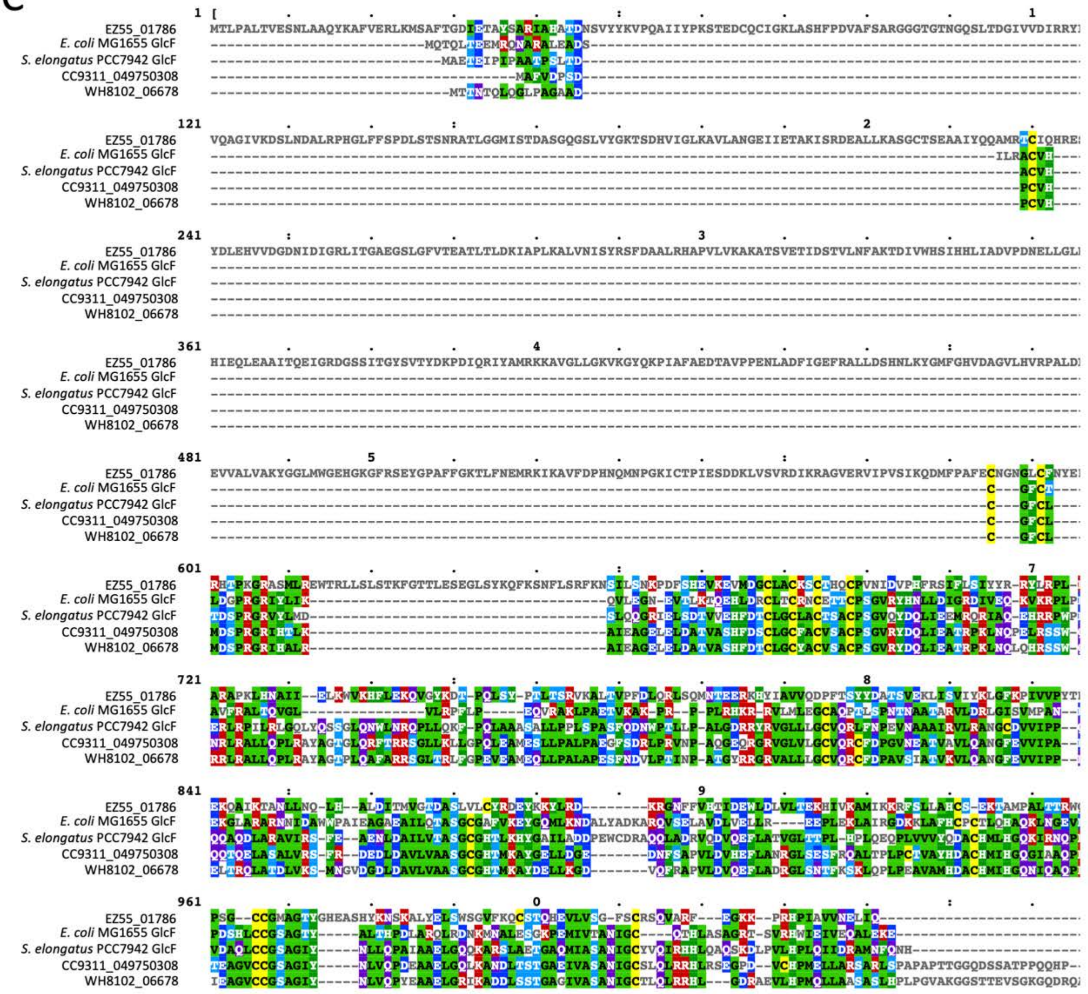
EZ55_01786
E. coli MG1655 GlcF
S. elongatus PCC7942 GlcF
CC9311_049750308
WH8102_06678

D

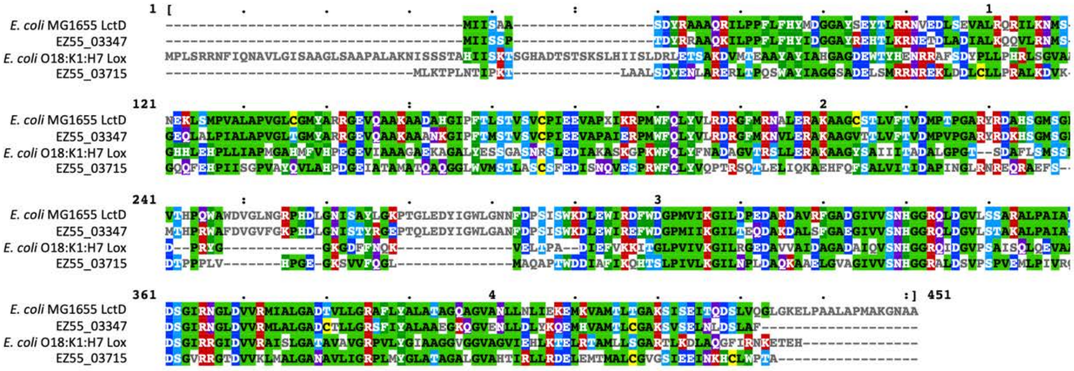
E. coli MG1655 LctD
EZ55_03347
E. coli O18:K1:H7 Lox
EZ55_03715

E

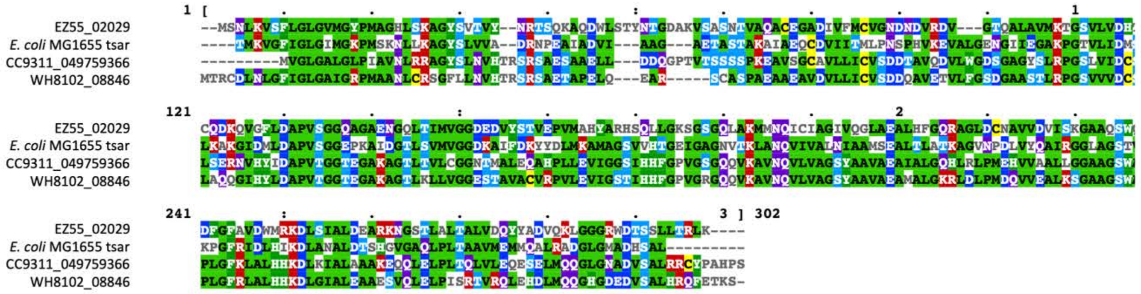

Figure S10. Alignment of hypothesized missing genes in the glycolate utilization pathways of the four strains in this study. Colored residues represent 70% consensus at the level of amino acid equivalence class. A-C) Candidates for the three subunits of bacterial glycolate dehydrogenase, GlcD, GlcE, and GlcF, respectively. For GlcD and GlcF, the putative *Alteromonas macleodii* EZ55 GlcDF fusion protein is included to show its homology with both enzymes. D) Putative lactate/glycolate oxidases. E) Putative tartronate semialdehyde reductases.

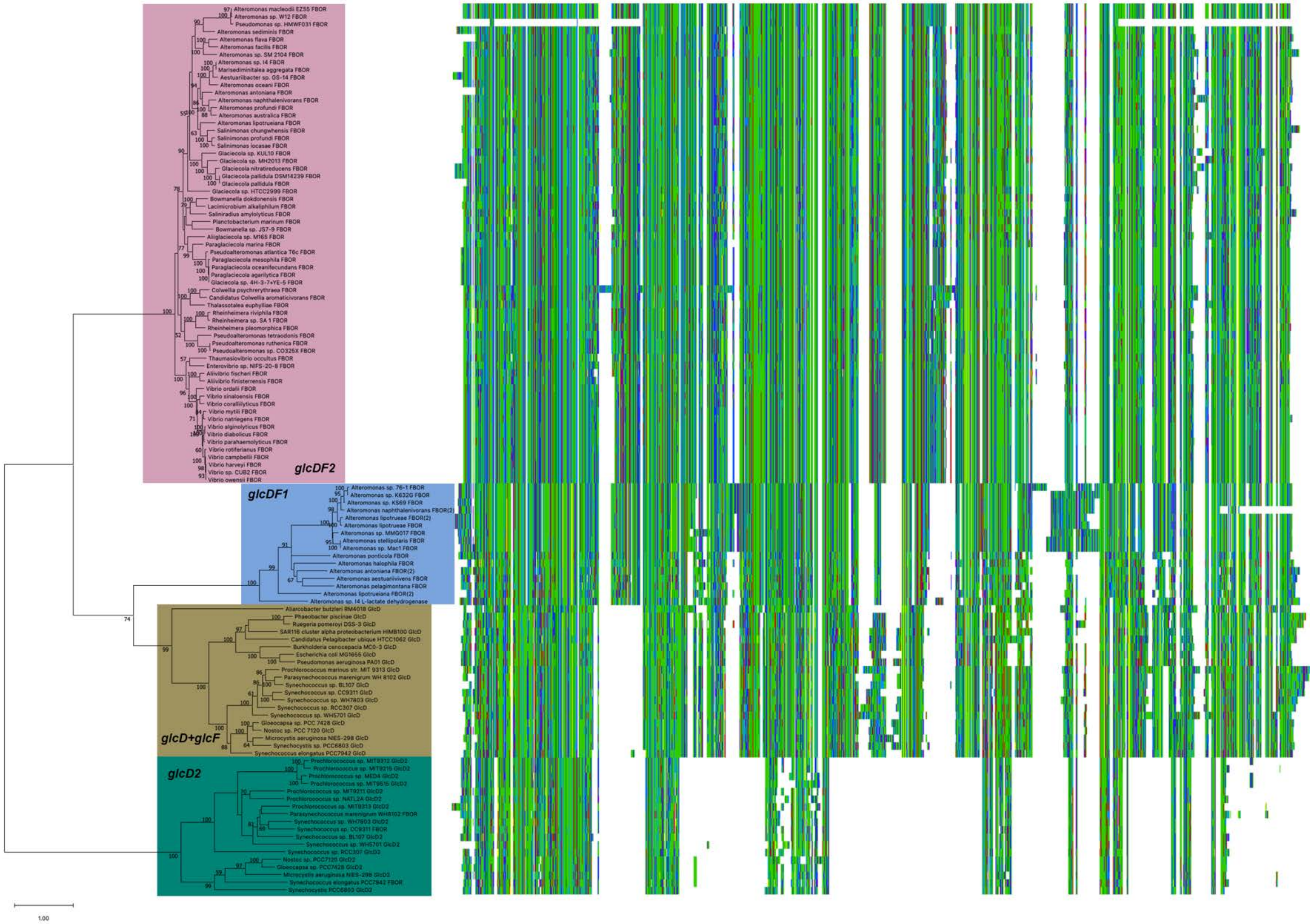

glcDF2
Alteromonas macleodii EZ55 FBOR
Alteromonas sp. W12 FBOR
Pseudomonas sp. HMWF031 FBOR
Alteromonas sediminis FBOR
Alteromonas flava FBOR
Alteromonas facilis FBOR
Alteromonas sp. SM 2104 FBOR
Alteromonas sp. I4 FBOR
Marisediminitalea aggregata FBOR
Aestuariibacter sp. GS-14 FBOR
Alteromonas oceani FBOR
Alteromonas antoniana FBOR
Alteromonas naphthalenivorans FBOR
Alteromonas profundi FBOR
Alteromonas australica FBOR
Alteromonas lipotrueiana FBOR
Salinimonas chungwhensis FBOR
Salinimonas profundi FBOR
Salinimonas iocasae FBOR
Glaciecola sp. KUL10 FBOR
Glaciecola sp. MH2013 FBOR
Glaciecola nitratireducens FBOR
Glaciecola pallidula DSM14239 FBOR
Glaciecola pallidula FBOR
Glaciecola sp. HTCC2999 FBOR
Bowmanella dokdonensis FBOR
Lacimicrobium alkaliphilum FBOR
Saliniradius amylolyticus FBOR
Planctobacterium marinum FBOR
Bowmanella sp. JS7-9 FBOR
Aliiglaciecola sp. M165 FBOR
Paraglaciecola marina FBOR
Pseudoalteromonas atlantica T6c FBOR
Paraglaciecola mesophila FBOR
Paraglaciecola oceanifecundans FBOR
Paraglaciecola agarilytica FBOR
Glaciecola sp. 4H-3-7+YE-5 FBOR
Colwellia psychrerythraea FBOR
Candidatus Colwellia aromaticivorans FBOR
Thalassotalea euphylliae FBOR
Rheinheimera riviphila FBOR
Rheinheimera sp. SA 1 FBOR
Rheinheimera pleomorphica FBOR
Pseudoalteromonas tetraodonis FBOR
Pseudoalteromonas ruthenica FBOR
Pseudoalteromonas sp. CO325X FBOR
Thaumasiovibrio occultus FBOR
Enterovibrio sp. NIFS-20-8 FBOR
Aliivibrio fischeri FBOR
Aliivibrio finisterrensis FBOR
Vibrio ordalii FBOR
Vibrio sinaloensis FBOR
Vibrio coralliilyticus FBOR
Vibrio mytili FBOR
Vibrio natriegens FBOR
Vibrio alginolyticus FBOR
Vibrio diabolicus FBOR
Vibrio parahaemolyticus FBOR
Vibrio rotiferianus FBOR
Vibrio campbellii FBOR
Vibrio harveyi FBOR
Vibrio sp. CUB2 FBOR
Vibrio owensii FBOR
glcDF1
Alteromonas sp. 76-1 FBOR
Alteromonas sp. K632G FBOR
Alteromonas sp. KS69 FBOR
Alteromonas naphthalenivorans FBOR(2)
Alteromonas lipotrueae FBOR(2)
Alteromonas lipotrueae FBOR
Alteromonas sp. MMG017 FBOR
Alteromonas stellipolaris FBOR
Alteromonas sp. Mac1 FBOR
Alteromonas ponticola FBOR
Alteromonas halophila FBOR
Alteromonas antoniana FBOR(2)
Alteromonas aestuariivivens FBOR
Alteromonas pelagimontana FBOR
Alteromonas lipotrueiana FBOR(2)
Alteromonas sp. I4 L-lactate dehydrogenase
glcD+glcF
Aliarcobacter butzleri RM4018 GlcD
Phaeobacter piscinae GlcD
Ruegeria pomeroyi DSS-3 GlcD
SAR116 cluster alpha proteobacterium HIMB100 GlcD
Candidatus Pelagibacter ubique HTCC1062 GlcD
Burkholderia cenocepacia MC0-3 GlcD
Escherichia coli MG1655 GlcD
Pseudomonas aeruginosa PA01 GlcD
Prochlorococcus marinus str. MIT 9313 GlcD
Parasynechococcus marenigrum WH 8102 GlcD
Synechococcus sp. BL107 GlcD
Synechococcus sp. CC9311 GlcD
Synechococcus sp. WH7803 GlcD
Synechococcus sp. RCC307 GlcD
Synechococcus sp. WH5701 GlcD
Gloeocapsa sp. PCC 7428 GlcD
Nostoc sp. PCC 7120 GlcD
Microcystis aeruginosa NIES-298 GlcD
Synechocystis sp. PCC6803 GlcD
Synechococcus elongatus PCC7942 GlcD
glcD2
Prochlorococcus sp. MIT9312 GlcD2
Prochlorococcus sp. MIT9215 GlcD2
Prochlorococcus sp. MED4 GlcD2
Prochlorococcus sp. MIT9515 GlcD2
Prochlorococcus sp. MIT9211 GlcD2
Prochlorococcus sp. NATL2A GlcD2
Prochlorococcus sp. MIT9313 GlcD2
Parasynechococcus marenigrum WH8102 FBOR
Synechococcus sp. WH7803 GlcD2
Synechococcus sp. CC9311 FBOR
Synechococcus sp. BL107 GlcD2
Synechococcus sp. WH5701 GlcD2
Synechococcus sp. RCC307 GlcD2
Nostoc sp. PCC7120 GlcD2
Gloeocapsa sp. PCC7428 GlcD2
Microcystis aeruginosa NIES-298 GlcD2
Synechococcus elongatus PCC7942 FBOR
Synechocystis PCC6803 GlcD2
1.00

Figure S11. Maximum likelihood tree of GlcDF-like proteins. Tree was constructed in MEGA 11 using WAG+G+I+F substitution model. Node values are the percentage support from 100 bootstrap replicates. *Micromonas pusilla* peroxisomal GOX was used as an outgroup to root the tree and was subsequently pruned for easier visualization. Colored patterns to the right of the tree show the protein alignments with mview color coding, indicating the strong conservation between the separate *glcD* and *glcF* genes previously studied and the *glcDF* fusion protein described here. FBOR, FAD-binding oxidoreductase.

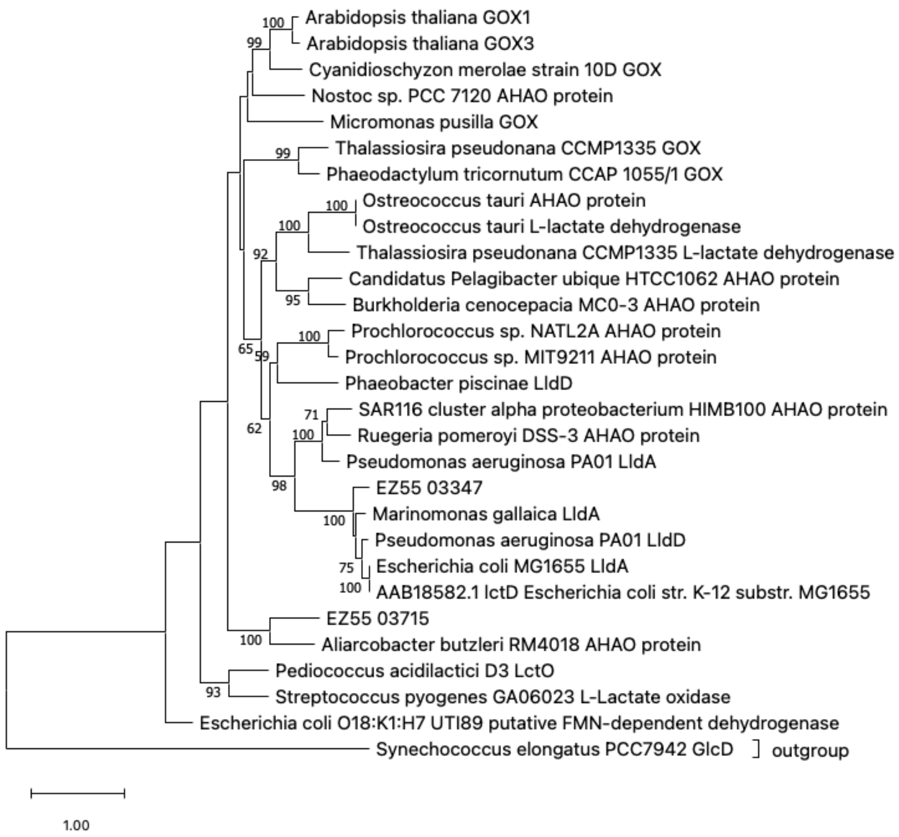

Figure S12. Maximum likelihood tree of LOX/GOX-like proteins. Tree was constructed in MEGA 11 using LG+G substitution model. Node values are the percentage support from 100 bootstrap replicates. *Synechococcus elongatus* glycolate dehydrogenase subunit GlcD was used as an outgroup to root the tree. AHAO, alpha-hydroxy-acid oxidizing
